# BRD9-mediated chromatin remodeling suppresses osteoclastogenesis through negative feedback mechanism

**DOI:** 10.1038/s41467-023-37116-5

**Published:** 2023-03-14

**Authors:** Jiahui Du, Yili Liu, Xiaolin Wu, Jinrui Sun, Junfeng Shi, Hongming Zhang, Ao Zheng, Mingliang Zhou, Xinquan Jiang

**Affiliations:** 1 Department of Prosthodontics, Shanghai Ninth People’s Hospital, Shanghai Jiao Tong University School of Medicine, Shanghai, 200011 China; 2 College of Stomatology, Shanghai Jiao Tong University, Shanghai, 200011 China; 3 National Center for Stomatology, National Clinical Research Center for Oral Diseases, Shanghai Key Laboratory of Stomatology, Shanghai Research Institute of Stomatology, Shanghai Engineering Research Center of Advanced Dental Technology and Materials, Shanghai, 200011 China

**Keywords:** Bone development, Disease model, Chromatin remodelling

## Abstract

Bromodomain-containing protein 9 (BRD9), a component of non-canonical BAF chromatin remodeling complex, has been identified as a critical therapeutic target in hematological diseases. Despite the hematopoietic origin of osteoclasts, the role of BRD9 in osteoclastogenesis and bone diseases remains unresolved. Here, we show *Brd9* deficiency in myeloid lineage enhances osteoclast lineage commitment and bone resorption through downregulating interferon-beta (IFN-β) signaling with released constraint on osteoclastogenesis. Notably, we show that BRD9 interacts with transcription factor FOXP1 activating *Stat1* transcription and IFN-β signaling thereafter. Besides, function specificity of BRD9 distinguished from BRD4 during osteoclastogenesis has been evaluated. Leveraging advantages of pharmacological modulation of BRD9 and flexible injectable silk fibroin hydrogel, we design a local deliver system for effectively mitigating zoledronate related osteonecrosis of the jaw and alleviating acute bone loss in lipopolysaccharide-induced localized aggressive periodontitis. Overall, these results demonstrate the function of BRD9 in osteoclastogenesis and its therapeutic potential for bone diseases.

## Abstract (web summary)

Osteoclast differentiation is critical for bone homeostasis. The authors show the negative feedback regulation of BRD9-mediated chromatin remodeling on osteoclastogenesis via interferon beta signaling and its therapeutic potential for bone diseases.

## Introduction

The function and homeostasis of bone relies on the balanced bone remodeling process, which is a continuously cycle of bone formation mediated by osteoblasts and resorption mediated by osteoclasts throughout life. The bone remodeling process is under sophisticatedly orchestrated with their imbalance can disturb the skeletal architecture and cause debilitating bone diseases such as osteoporosis, osteopetrosis, osteonecrosis, Paget’s disease and so on^1^.

Osteoclasts are hematopoietic myeloid lineage and derived from bone marrow-derived monocytes (BMDMs). In the last decades, it is quite well-documented that osteoclastogenesis is initiated with receptor activator of nuclear factor κB (NF-κB) ligand (RANKL) and macrophage colony stimulating factor (M-CSF) in the bone microenvironment^2^. Afterward, it is followed by a series of activation, proliferation, fusion and maturation process, then the terminally differentiated multinucleated osteoclasts are able to secrete proteases and acids, such as tartrate resistant acid phosphatase (TRAP), cathepsin K (CTSK) and matrix metallopeptidase 9 (MMP9), conducting their bone resorptive activity^3^.

The above osteoclast lineage commitment and differentiation are sophisticatedly regulated by complex signal transduction cascades that converge to transcriptional modulation^4^. Mammalian BRG1-associated factors (BAF) complex is ATP-dependent chromatin remodelers comprising multiple protein subunits that translocate nucleosome and regulate gene transcription with DNA-binding factors^5,6^. Multiple lines of evidence indicate that the BAF complex plays essential roles for controlling expression of genes linked to hematopoietic developmental processes at promoter and enhancer regions, alongside its better-known function in suppressing a variety of cancers in humans^5,7,8^. In particular, bromodomain-containing protein 9 (BRD9), a crucial component of the distinct non-canonical BAF (ncBAF) complex, was identified to exert a prominent function during myeloid lineage development^9–11^. For example, acute myeloid leukemia appears to be specific-dependent on the BRD9-containing BAF complex via the activation of STAT5 pathway^11^. By targeting BRD9, AML cells undergo cell cycle arrest and differentiate, suggesting BRD9 as an essential therapeutic target in leukemia^10^. Besides, it has been established that BRD9 regulates interferon-stimulated genes (ISGs) during macrophage activation via cooperation with BET protein BRD4 and also acts a modulator of glucocorticoid responses^12,13^. These observations suggested that BRD9 is crucial during myeloid lineage determination as well as macrophage inflammatory response. Osteoclasts are myeloid origin and share the common ancestor with macrophage in the hematopoietic lineage. However, there was little evidence for the association between BRD9-mediated chromatin remodeling and osteoclast lineage differentiation in bone homeostasis.

Here, we report that the gene expression of BRD9 is upregulated during RANKL-induced osteoclast differentiation. *Brd9* deficiency in myeloid lineage enhanced osteoclast lineage commitment and bone resorption through downregulating interferon-beta (IFN-β) signaling with released constraint on osteoclastogenesis. Notably, we show that BRD9 interacts with transcription factor (TF) FOXP1 and activates *Stat1* transcription and IFN-β signaling thereafter. Besides, function specificity of BRD9 distinguished from BRD4 during osteoclastogenesis were evaluated. Leveraging the advantages of pharmacological modulation of BRD9 and flexible injectable silk fibroin hydrogel, we designed a local deliver system for effectively mitigating zoledronate (ZOL)-related osteonecrosis of the jaw (ONJ) and acute bone loss in lipopolysaccharide (LPS)-induced localized aggressive periodontitis. In summary, these findings indicate that BRD9-FOXP1-STAT1 axis is crucial for the negative feedback signaling in osteoclastogenesis, expand our knowledge of the intricate interactions among chromatin remodelers, transcription factors, and signaling pathways in bone homeostasis and also highlight its therapeutic potential for bone diseases.

## Results

### Loss of *Brd9* in myeloid lineage leads to less bone mass

**Fig. 1:**
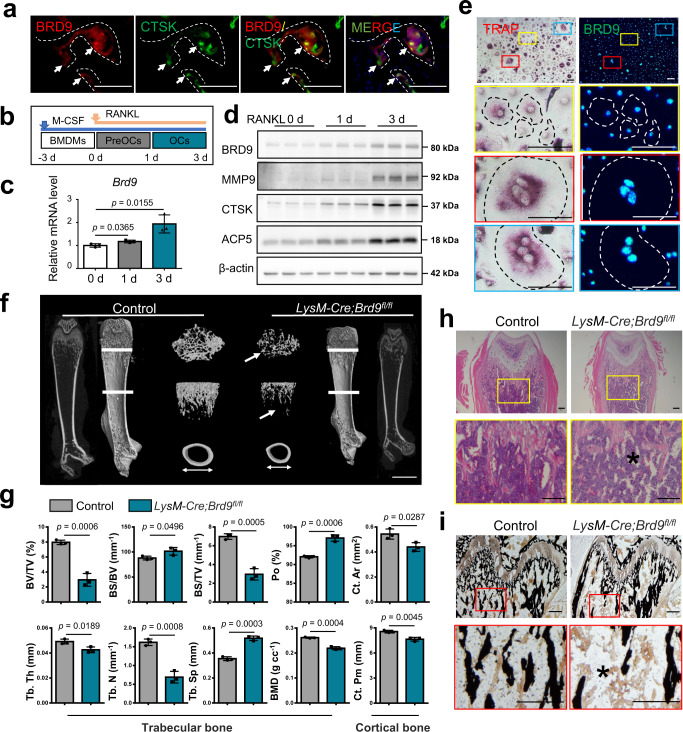
Loss of *Brd9* in myeloid lineage leads to less bone mass. **a** Immunofluorescence staining of BRD9 (red) and CTSK (green) in the mouse distal femur at 4 weeks of age. White dashed lines outline the surface of trabecular bone. Scale bar, 100 μm. **b** Schema for osteoclastic differentiation stages of BMDMs in vitro. Bone marrow-derived monocytes, BMDMs, osteoclast precursors, preOCs, and matured osteoclast, OCs. **c** *Brd9* mRNA expression in BMDMs at indicated days after RANKL-induction, as measured by qPCR. *n* = 3 biologically independent samples. **d** BRD9, MMP9, CTSK and ACP5 protein expression in BMDMs at indicated days after RANKL-induction. **e** Immunofluorescence staining of BRD9 (green) and TRAP staining (red) in BMDMs after 3 days of RANKL-induction. Colored boxes in the first panel are shown at higher magnification in below. Dashed lines outline the cell boundaries. Scale bar, 100 μm. **f** Representative micro-CT image of the distal trabecular bone and cortical bone of femurs from 4-week-old *LysM-Cre;Brd9*^*fl/fl*^ mice and littermate control mice. Arrows indicates the decreased bone mass. Scale bar, 2 mm. **g** Quantification analysis of bone volume/tissue volume ratio (BV/TV), bone surface/volume ratio (BS/BV), bone volume/tissue volume ratio (BS/TV), porosity percent (Po), trabecular thickness (Tb. Th), trabecular number (Tb. N), trabecular separation (Tb. Sp), bone mineral density (BMD), cortical crossectional bone area (Ct. Ar) and perimeter (Ct. Pm) of femurs from 4-week-old *LysM-Cre;Brd9*^*fl/fl*^ mice and littermate control mice. *n* = 3. **h** H&E staining of femurs from 4-week-old *LysM-Cre;Brd9*^*fl/fl*^ mice and littermate control mice. Box in the first panel is shown at higher magnification in below. Asterisk indicates the decreased bone mass. Scale bar, 200 μm. **i** Von Kossa staining of femurs from 4-week-old *LysM-Cre;Brd9*^*fl/fl*^ mice and littermate control mice. Box in the first panel is shown at higher magnification in below. Asterisk indicates the decreased bone mineralization. Scale bar, 200 μm. All data in this figure are represented as mean ± SD. Two-tailed Student’s *t*-test for (**c**) and (**g**). All experiments were performed in triplicates unless otherwise stated. Source data are provided in the Source data file.

To investigate the critical role of BRD9 in osteoclastogenesis, we examined its expression pattern in osteoclast lineage cell. We found that BRD9 is high expressed in CTSK + osteoclasts on the surface of trabecular bone of distal femur from the 4-week-old mouse (Fig. 1a). Moreover, BMDMs were cultured with M-CSF and RANKL to further evaluate the expression level of BRD9 during proliferation, differentiation, and maturation stages at indicated days (Fig. 1b). We found that BRD9 expression was gradually upregulated after RANKL treatment both at mRNA and protein level during the osteoclastogenesis (Fig. 1c, d), which is also well visualized in the immunofluorescent staining of BRD9 in BMDMs after RANKL-induction in vitro (Fig. 1e).

To further determine whether BRD9 plays a crucial role during osteoclastogenesis, we generated knockout mice through specific deletion of BRD9 in the osteoclast lineage. Mice bearing *loxP* sites encompassing the *Brd9 exon6-exon7* (*Brd9*^*fl/fl*^ mice) were crossed with a myeloid-specific *Cre*-recombinase (*Cre*)-expressing mouse line, those expressing Cre driven by the lysozyme M promoter (*LysM-Cre* mice). Mice lacking *Brd9* in *LysM*-Cre-expressing cells, hereafter referred to as *LysM-Cre;Brd9*^*fl/fl*^ mice (Supplementary Fig. 1a). It showed that BRD9 were knocked out efficiently at both mRNA and protein levels in BMDMs from *LysM-Cre;Brd9*^*fl/fl*^ mice (Supplementary Fig. 1b, c).

In μCT analysis, *LysM-Cre;Brd9*^*fl/fl*^ mice exhibited decreased femoral trabecular bone mass compared to control mice at 4 weeks of age, with decreased bone volume/tissue volume ratio (BV/TV), bone volume/tissue volume ratio (BS/TV), trabecular thickness (Tb.Th), trabecular number (Tb.N), bone mineral density (BMD) and increased bone surface/volume ratio (BS/BV), trabecular separation (Tb.Sp) and porosity percent (Po). *LysM-Cre;Brd9*^*fl/fl*^ mice also exhibited decreased cortical thickness at 4 weeks of age, with decreased cross sectional bone area (Ct.Ar) and perimeter (Ct.Pm) (Fig. 1f, g). Histologically, the H&E staining (Fig. 1h) and Von Kossa staining (Fig. 1i) results showed the decreased bone mass and mineralization in the distal femurs after *Brd9* loss in myeloid cell lineage compared with littermate control mice at 4 weeks of age.

### *Brd9* deletion enhances osteoclast lineage commitment in vivo

**Fig. 2:**
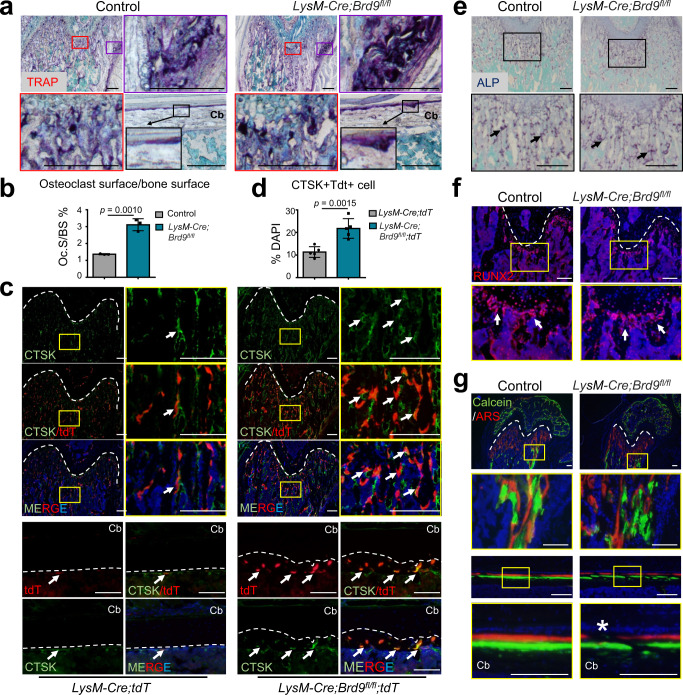
Loss of *Brd9* in myeloid lineage leads to enhanced osteoclast lineage commitment in vivo. **a** TRAP staining of distal trabecular and cortical bone of femurs from 4-week-old *LysM-Cre;Brd9*^*fl/fl*^ mice and littermate control mice. Colored box in the top left corner is shown at higher magnification in right and below. Cortical bone, Cb. Scale bar, 200 μm. **b** Quantitative analysis of the TRAP + osteoclast surface/bone surface ratios (Oc.S/BS) in the femurs from 4-week-old *LysM-Cre;Brd9*^*fl/fl*^ mice and littermate control mice. *n* = 3. **c** CTSK immunofluorescence (green) and visualization of tdTomato (red) of femurs from 4-week-old *LysM-Cre;Brd9*^*fl/fl*^*;tdT* mice and littermate control mice. Box in the left is shown at higher magnification in right. The progeny of the myeloid lineage shows red signal. Arrows indicate double positive signals. White dashed lines outline the growth plate of distal femur. Cortical bone, Cb. Scale bar, 200 μm. **d** Quantitative analysis of the ratio of CTSK + tdT+ myeloid lineage osteoclasts in the femurs from 4-week-old *LysM-Cre;Brd9*^*fl/fl*^*;tdT* mice and littermate control mice. *n* = 5. **e** ALP staining (purple) of distal femur from *LysM-Cre;Brd9*^*fl/fl*^ mice and littermate control mice at 4 weeks of age. Arrows indicate positive signals. Scale bar, 200 μm. **f** RUNX2 immunofluorescence (red) of distal femur from *LysM-Cre;Brd9*^*fl/fl*^ mice and littermate control mice at 4 weeks of age. Arrows indicate RUNX2-positive osteoblasts. White dashed lines outline the growth plate of distal femur. Scale bar, 200 μm. **g** visualization of calcein/ Alizarin Red S (ARS) labeling in *LysM-Cre;Brd9*^*fl/fl*^ mice and littermate control mice at age of 4 weeks. Asterisk indicates the eroded bone surface. White dashed lines outline the growth plate of distal femur. Cortical bone, Cb. Scale bar, 200 μm. All data in this figure are represented as mean ± SD. Two-tailed Student’s *t*-test for (**b**) and (**d**). All experiments were performed in triplicates unless otherwise stated. Source data are provided in the Source data file.

To investigate the cellular mechanism accounting for the decreased bone mass after *Brd9* loss in myeloid cell lineage, we examined osteoclastic bone resorption and osteoblastic bone formation individually. We found that the number and size of TRAP-positive differentiated osteoclasts were increased on the bone surface of the femurs from *LysM-Cre;Brd9*^*fl/fl*^ mice at 4 weeks of age (Fig. 2a, b). Furthermore, using Tdt as a reporter to trace the myeloid cell fate, we found that the cell number of CTSK + osteoclasts from *LysM* + myeloid lineage (Tdt+CTSK + cell) was increased apparently on the bone surface of the femurs in the mutant mice compared to control mice at 4 weeks of age, suggesting enhanced osteoclast lineage commitment and bone resorption after loss of *Brd9* (Fig. 2c, d).

To determine if the osteoblastic bone formation is also affected in *LysM-Cre;Brd9*^*fl/fl*^ mice, we investigate the expression of alkaline phosphatase (ALP) (Fig. 2e) and the number of RUNX2 + osteoblasts on the trabecular bone surface at 4 weeks of age (Fig. 2f). It was showed that the number of osteoblasts and osteoblastic osteogenic activity was comparable between *LysM-Cre;Brd9*^*fl/fl*^ mice and littermate control mice. We then assessed the mineral apposition on the bone surface dynamically with calcein/Alizarin Red S (ARS) labeling. The results showed that the bone formation rate was comparable while more eroded bone surface was observed in *LysM-Cre;Brd9*^*fl/fl*^ mice compared with littermate control mice at 4 weeks of age (Fig. 2g).

Thus, it is conceivable that the enhanced osteoclast lineage commitment following *Brd9* ablation accelerates bone resorption, leading to excessive bone mass loss.

### BRD9 suppresses RANKL-induced osteoclast differentiation in vitro

**Fig. 3:**
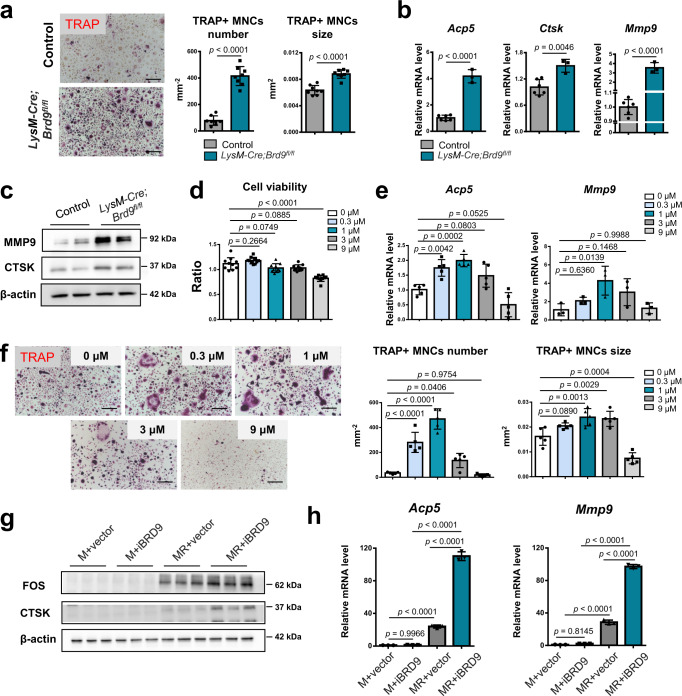
BRD9 deletion and inhibition in BMDMs leads to overactivated osteoclastogenesis in vitro. **a** TRAP staining and quantification analysis of BMDMs from 4-week-old *LysM-Cre;Brd9*^*fl/fl*^ mice and littermate control mice after 3 days of RANKL-induction. Scale bar, 200 μm. *n* = 8. **b** The mRNA expression of osteoclastic-specific genes of *Acp5*, *Ctsk,* and *Mmp9* in BMDMs from *LysM-Cre;Brd9*^*fl/fl*^ mice compared with that from control littermates after 3 days of RANKL-induction, as measured by qPCR. *n* = 3 for *LysM-Cre;Brd9*^*fl/fl*^ mice group. *n* = 6 for control littermates. **c** The protein expression of MMP9 and CTSK in BMDMs from *LysM-Cre;Brd9*^*fl/fl*^ mice compared with that from control littermates after 3 days of RANKL-induction, as measured by western blot. **d** Cell viability of RANKL-induced BMDMs with 1 day of iBRD9 at different concentration, shown by cell counting kit 8 assay. *n* = 10 biologically independent samples. **e** The mRNA expression of *Acp5* and *Mmp9* in RANKL-induced BMDMs with 1 day of iBRD9 at different concentration, as measured by qPCR. *n* = 5 biologically independent samples for *Acp5*. *n* = 3 biologically independent samples for *Mmp9*. **f** TRAP staining and quantification analysis of RANKL-induced BMDMs with 5 days of iBRD9 at different concentration. Scale bar, 200 μm. *n* = 5 biologically independent samples. **g** The protein expression of FOS and CTSK in BMDMs after 3 days of 1 μM iBRD9 or vector treatment with or without RANKL-induction, as measured by western blot. M-CSF, M; RANKL, R. **h** The mRNA expression of *Acp5* and *Mmp9* in BMDMs after 3 days of 1 μM iBRD9 or vector treatment with or without RANKL-induction, as measured by qPCR. M-CSF, M; RANKL, R. *n* = 3 biologically independent samples. All data in this figure are represented as mean ± SD. Two-tailed Student’s *t*-test for (**a**) and (**b**). One-way analysis of variance (ANOVA) with Dunnett’s multiple comparisons test for (**d**), (**e**), and (**f**); ANOVA with Tukey’s multiple comparisons test for (**h**). All experiments were performed in triplicates unless otherwise stated. Source data are provided in the Source data file.

Furthermore, the BMDMs from *LysM-Cre;Brd9*^*fl/fl*^ and control mice at 4 weeks of age were administered with RANKL for osteoclastic induction in vitro. In line with the role of BRD9 in vivo, we found that an increased number and larger size of TRAP + multinuclear cells from *Brd9* depleted BMDMs than that from control BMDMs after 3 days of RANKL-induction (Fig. 3a). And the expression of osteoclastic-specific genes of *Acp5*, *Ctsk,* and *Mmp9* were increased in BMDMs from *LysM-Cre;Brd9* ^*fl/fl*^ mice compared with that from control littermates (Fig. 3b, c).

The function of BRD9 during osteoclastogenesis is also investigated with a selective cellular chemical inhibitor for BRD9, iBRD9^14,15^. IBRD9 treatment in BMDMs leads to dose-dependent increase on the gene expression of *Acp5* and *Mmp9* in osteoclastic induced BMDMs after 24 h, without apparent cell toxicity under the concentration of 9 μM (Fig. 3d, e). Consistently, we also confirmed the dose-dependent increase on the number and size of TRAP + multinuclear differentiated osteoclasts from RANKL-induced BMDMs with iBRD9 treatment (Fig. 3f). The concentration of 1 μM iBRD9 was chosen in the further studies for its most prominent function on promoting osteoclastogenesis.

To further investigate whether BRD9 inhibitor could induce osteoclastogenesis alone or function under RANKL-induction, we compared the protein expression of FOS, CTSK, as well as the mRNA expression of *Acp5* and *Mmp9* in BMDMs after 1 μM iBRD9 or vector treatment with or without RANKL-induction. We found that BRD9 inhibitor exert no apparent effect on osteoclastogenesis without RANKL-induction, suggesting the function of BRD9 is RANKL-dependent (Fig. 3g, h).

Taken together, these findings suggest that BRD9, which was upregulated after RANKL treatment, exerts suppressing regulation on RANKL-induced osteoclast differentiation in a negative feedback mechanism, and both genetic deletion and inhibition of BRD9 in BMDMs consistently promotes osteoclast differentiation.

### BRD9 suppresses osteoclastogenesis via interferon-beta signaling

**Fig. 4:**
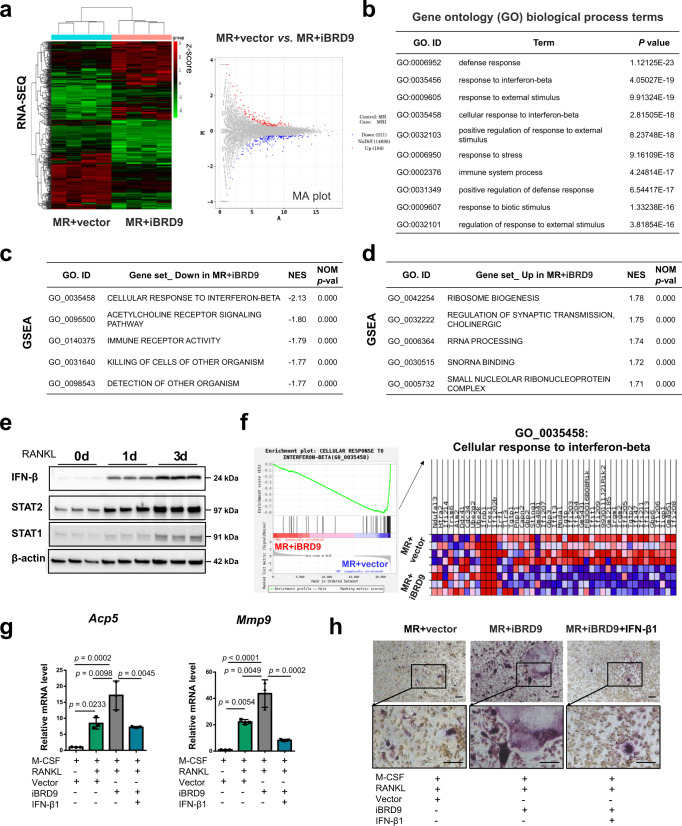
BRD9-mediated transcriptional activation of interferon-beta signaling negative regulates osteoclastogenesis. **a** Heatmap hierarchical clustering and the MA plot showing the gene expression profiles in BMDMs after 1 day of BRD9 inhibition during osteoclastic differentiation with M-CSF and RANKL (MR). *n* = 4. Color scale represents normalized gene FPKM value by z-score scheme. **b** The top 10 Gene ontology (GO) biological process enriched in the comparison between BMDMs in MR + vector and MR + iBRD9 group**. c** Gene set enrichment analysis (GSEA) analysis of the top downregulated gene sets in MR + iBRD9 group compared with control group. **d** GSEA analysis of the top upregulated gene sets in MR + iBRD9 group compared with control group. **e** The protein expression of IFN-β, STAT2, and STAT1 in BMDMs at different days after osteoclastic differentiation, as measured by western blot. **f** GSEA plots and heatmap hierarchical clustering of GO term_0035458 in MR + iBRD9 group and control group. *n* = 4. **g** The mRNA expression of *Acp5* and *Mmp9* in BMDMs treated with 2 days of 1 μM iBRD9 and 0.0625 ng/ml IFN-β1 during osteoclastic induction, as measured by qPCR. *n* = 3 biologically independent samples. **h** TRAP staining of BMDMs treated with 3 days of 1 μM iBRD9 and 0.0625 ng/ml IFN-β1 during osteoclastic induction. Scale bar, 200 μm. All data in this figure are represented as mean ± SD. Hypergeometric distribution test for (**b**). Empirical phenotype-based permutation test for (**c**) and (**d**). One-way analysis of variance (ANOVA) with Tukey’s multiple comparisons test for (**g**). All experiments were performed in triplicates unless otherwise stated. Source data are provided in the Source data file.

To further investigate the feedback mechanism of BRD9-mediated suppression on RANKL-induced osteoclast differentiation, we conducted mRNA sequencing to compare the transcription profile in BMDMs after 1 day of BRD9 inhibition during osteoclastic differentiation with M-CSF and RANKL, refer as MR + iBRD9, with the control group (MR + vector). The RNA-sequencing data showed well-separated gene expression patterns between two groups with a total of 211 downregulated and 184 upregulated genes in BMDMs in MR + iBRD9 group (fold change > = 1.2; *p* < 0.05), as shown in the heatmap and MA plot (Fig. 4a). The 10 top gene ontology (GO) biological process terms in the comparison between BMDMs in MR + vector and MR + iBRD9 group, are intensely enriched on defense response, response to interferon-beta (IFN-β) and external stimulus, and immune system process and their topological relationships illustrated in a directed acyclic graph (DAG) (Fig. 4b and Supplementary Fig. 2). Gene set enrichment analysis (GSEA) further revealed that the IFN-β response, acetylcholine receptor signaling, and immune receptor activity were significantly downregulated, while ribosome biogenesis, synaptic transmission, and rRNA processing were upregulated in MR + iBRD9 group (Fig. 4c, d).

Thereinto, IFN-β signaling activity, as one of the top downregulated signaling pathways after BRD9 inhibition, is reported to activate ISGs through interferon-stimulated gene factor 3 (ISGF3), which consists of activated signal transducer and activator of transcription 1 (STAT1), signal transducer and activator of transcription 2 (STAT2), and interferon regulatory factors^16^. Previous studies have shown that global IFN-β-knockout mice display an osteoporotic phenotype due to an excessive osteoclastogenesis^17^. In our present study, we identified that RANKL activates the IFN-β signaling with increased IFN-β, STAT1, STAT2 during osteoclastogenesis (Fig. 4e), which synchronizes with the expression profile of BRD9 (Fig. 1c, d). While IFN-β signaling activity was downregulated in RANKL-induced BMDMs after iBRD9 treatment (Fig. 4f). Therefore, we presumed that the transcriptional downregulated IFN-β signaling in BMDMs may, at least partially, contribute to the excessive osteoclastogenesis after BRD9 inhibition. To verify this hypothesis, we added IFN-β1 cytokine into BRD9 inhibited BMDMs, and found that the increased osteoclastogenesis after BRD9 inhibition was rescued after IFN-β signaling upregulation, as shown with the downregulated expression of osteoclastogenic genes of *Acp5* and *Mmp9* (Fig. 4g), and the decreased number and size of TRAP + differentiated osteoclasts (Fig. 4h).

Since it is reported that glucocorticoid/dexamethasone (DEX) promotes osteoclastogenesis^18^, and BRD9 inhibition attenuated macrophage inflammation by synergizing with glucocorticoid response^13^, the potential synergizing function of BRD9 inhibition and glucocorticoid signaling during osteoclastogenesis has been further analyzed. The results have shown that DEX promotes osteoclastogenesis at the concentration of 10^−9 ^M, with no significant promotion below the concentration of 10^−10 ^M. While the low DEX dose showed promotion on osteoclastogenesis when combined with iBRD9 at 0.1 μM, the low concentration exerting no apparent effect on osteoclastogenesis alone (Supplementary Fig. 3a, b). Then we conducted mRNA sequencing analysis to unveil the change on gene transcription profile after synergistic 0.1 μM iBRD9/10^−10 ^M DEX treatment during osteoclastogenesis. Besides of the apparent activation on osteoclast-related genes (Supplementary Fig. 3c), the enriched top ten changed signaling pathways in iBRD9/DEX-treated group compared with the control group were listed (Supplementary Fig. 3d). We noticed the functional categories of iBRD9/DEX cooperatively regulated genes were enriched on metabolic process, electron transport chain, and peptide biosynthetic process during osteoclastogenesis. The regulatory mechanisms differ from 1 μM iBRD9-alone regulated gene network, which enriched in response to IFN-β signaling and external stimulus mentioned above, and also differ from the finding in macrophage activation that the synergistic gene signature is enriched in inflammatory responses categories^13^. This finding suggests that BRD9 modulated regulatory mechanism is characterized with context-dependency and cell type-specificity.

In summary, we conclude that the transcriptional activation on IFN-β signaling in BMDMs may contribute to BRD9-mediated negative feedback during osteoclastogenesis.

### *Stat1* is critical for BRD9-mediated osteoclastogenesis suppression

To further elucidate how BRD9 participates in the transcriptional regulation during the negative feedback of RANKL-induced osteoclastogenesis, we compared RANKL-induced, iBRD9-inhibited gene transcription with the binding profile of BRD9 in BMDMs during osteoclastogenesis using immunoprecipitation (ChIP) sequencing analysis.

**Fig. 5:**
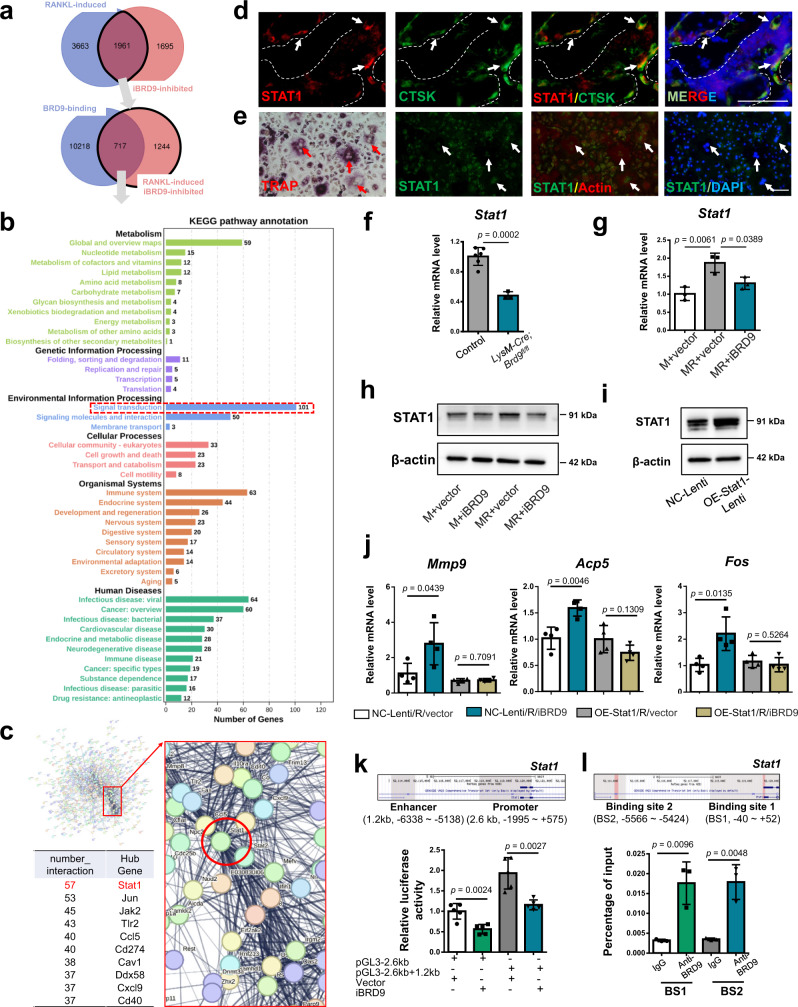
STAT1 is critical for BRD9-mediated negative feedback during osteoclastogenesis. **a** Venn diagram showing overlapping genes, where BRD9 binding, RANKL-induced while downregulated after BRD9 inhibition. **b** KEGG pathway annotation of the genes, where BRD9 binding, RANKL-induced while downregulated after BRD9 inhibition. **c** Protein-protein interaction (PPI) networks of the genes, where BRD9 binding, RANKL-induced while downregulated after BRD9 inhibition. **d** Immunofluorescence staining of STAT1 (red) and CTSK (green) in the mouse distal femur at 4 weeks of age. Scale bar,100 μm. **e** Immunofluorescence of STAT1 (green), TRAP staining (red) and actin (red) in BMDMs after 3 days of RANKL-induction. Scale bar, 100 μm. **f** The mRNA expression of *Stat1* in BMDMs from *LysM-Cre;Brd9*^*fl/fl*^ mice compared with that from control littermates after 3 days of RANKL-induction. *n* = 3 for *LysM-Cre;Brd9*^*fl/fl*^ mice group. *n* = 6 for control littermates. **g** The mRNA expression of *Stat1* in BMDMs treated with iBRD9 after 2 days of RANKL-induction. *n* = 3 biologically independent samples. **h** The protein expression of STAT1 in BMDMs treated with iBRD9 after 2 days of RANKL-induction. **i** The protein expression of STAT1 in RAW264.7 transfected with control lentivirus (NC) and *Stat1*-overexpression lentivirus (OE-Stat1). **j** The mRNA expression of *Mmp9*, *Acp5* and *Fos* in *Stat1*-overexpressed and control RAW264.7 treated with iBRD9 after 2 days of RANKL-induction. *n* = 4 biologically independent samples. **k** Luciferase reporter activities of the *Stat1* promoter alone or in the presence of enhancer region in osteoclastic induced RAW264.7 treated with iBRD9 or control vector. *n* = 5 biologically independent samples. **l** ChIP assay with BRD9 antibody (or IgG) in RAW264.7 cell line during osteoclastic induction. *n* = 3 biologically independent samples. All data in this figure are represented as mean ± SD. Two-tailed Student’s *t*-test for (**f**), (**j**), (**k**), and (**l**). One-way analysis of variance (ANOVA) with Tukey’s multiple comparisons test for (**g**). M, M-CSF; MR, M-CSF + RANKL. All experiments were performed in triplicates unless otherwise stated. Source data are provided in the Source data file.

There are 717 genes, where BRD9 binding, RANKL-induced while downregulated after BRD9 inhibition (Fig. 5a). KEGG enrichment analysis of above genes unveiled that the critical transcriptional regulations of BRD9 were enriched on signal transduction, signaling molecules, and interaction as well as immune response (Fig. 5b). The protein-protein interaction (PPI) analysis further identified several crucial hub genes among the gene list, suggesting their potential direct downstream targets of BRD9 during the negative feedback regulation during osteoclastogenesis (Fig. 5c). Thereinto, we noticed STAT1, as one of the hub genes with most interaction numbers and a primary mediator for IFN-β signaling activity^19^, is increased after RANKL-induction and expressed in osteoclasts in vivo and in vitro (Figs. 4e, 5d, e). While the gene expression of *Stat1* was downregulated in BMDMs from *LysM-Cre;Brd9*^*fl/fl*^ mice compared with that from control littermates after RANKL-induction (Fig. 5f) and also downregulated in BMDMs from wild-type mice after BRD9 inhibition (Fig. 5g and h). Previous studies have identified that STAT1, as an important factor involved in osteoporosis, is importance for bone metabolism^19,20^. It has been observed that excessive osteoclastogenesis existed in the bone of the *Stat1*-deficient mice, suggesting its negative regulation of osteoclast differentiation^21^. Therefore, we hypothesized that Stat1 is one of the critical downstream targets in BRD9-mediated negative feedback during osteoclastogenesis.

Mouse macrophage cell line RAW264.7 is a preferred model for molecular mechanism study on osteoclastogenesis^22^. Therefore, we constructed the STAT1 constituently expressed RAW264.7 cell line using lentivirus vector for further function test of iBRD9 inhibitor (Fig. 5i). We found that the expression of osteoclastic-related genes, such as *Mmp9* and *Acp5*, were comparable between control and iBRD9 inhibitor treated *Stat1*-overexpressed RAW264.7 cells, while increased apparently in control-lentivirus transfected RAW264.7 cells after BRD9 inhibition (Fig. 5j). These findings suggest that the downregulated expression of *Stat1* might be a key process in BRD9 inhibition mediated excessive osteoclastogenesis.

Previous studies have indicated that BAF complex plays essential roles for controlling expression of genes linked to developmental processes at promoter and enhancer regions^7,9^. It is also reported that the 5.5 kb upstream regulatory region of the mouse *Stat1* gene enriches with similar epigenetic signatures with proximal promoter region, functioning as a transcriptional enhancer and conserved among mammals^23^. To further validate the transcriptional regulation of BRD9 on *Stat1*, we cloned the predicted proximal promoter (2.6 kb, −1995 ~ +575) and the enhancer (1.2 kb, −6338 ~ −5138) region of *Stat1* into the PGL3-basic vector to drive the expression of luciferase reporter (Supplementary Fig. 4). We found that the *Stat1* proximal promoter construct in the presence of upstream enhancer region exhibited greater reporter activities than the *Stat1* proximal promoter alone, while BRD9 inhibition repressed the transcription activity of both *Stat1* promoter and enhancer apparently (Fig. 5k). Then, we conducted ChIP-qPCR in osteoclastic induced RAW264.7 cells and validated BRD9 could bind to the promoter and the enhancer of *Stat1* directly (Fig. 5l, Supplementary Fig. 4).

Mutations in *STAT1* gene have been shown to be in association with multifocal osteomyelitis in patients with Mendelian susceptibility to mycobacterial disease (MSMD), due to impaired inhibition of osteoclast differentiation and bone resorption^24,25^. We also detected there are multiple protein-altering variants, splice site variants and non-coding transcript or untranslated region variants in above predicted promoter and enhancer region of *Stat1*, from the European Variation Archive Release 3, as generated by the UCSC Genome Browser (Supplementary Fig. 5). The finding of enriched nucleotide variants near the binding sites of BRD9 at *Stat1*, further enlightens that the critical epigenetic regulation of BRD9 relates to human disease.

Collectively, all these results demonstrate that BRD9 promotes *Stat1* transcription by directly regulating its promoter and enhancer activity.

### FOXP1 interacts with BRD9 on *Stat1* transcriptional regulation

It is well-documented that chromatin remodeler regulates tissue- and stage-specific gene transcription with the facilitation of cofactors containing DNA sequence-specific binding functions^26,27^. As a critical component of the ncBAF complex, the biological roles of BRD9 largely depend on the collaborating TFs and the specific context^11,13,28^. To investigate the chromatin remodeling function of BRD9 and cofactors possibly facilitating the negative action of BRD9 on osteoclastogenesis, we conducted the Assay for Transposase-Accessible Chromatin with high throughput sequencing (ATAC-seq) and motif analysis.

**Fig. 6:**
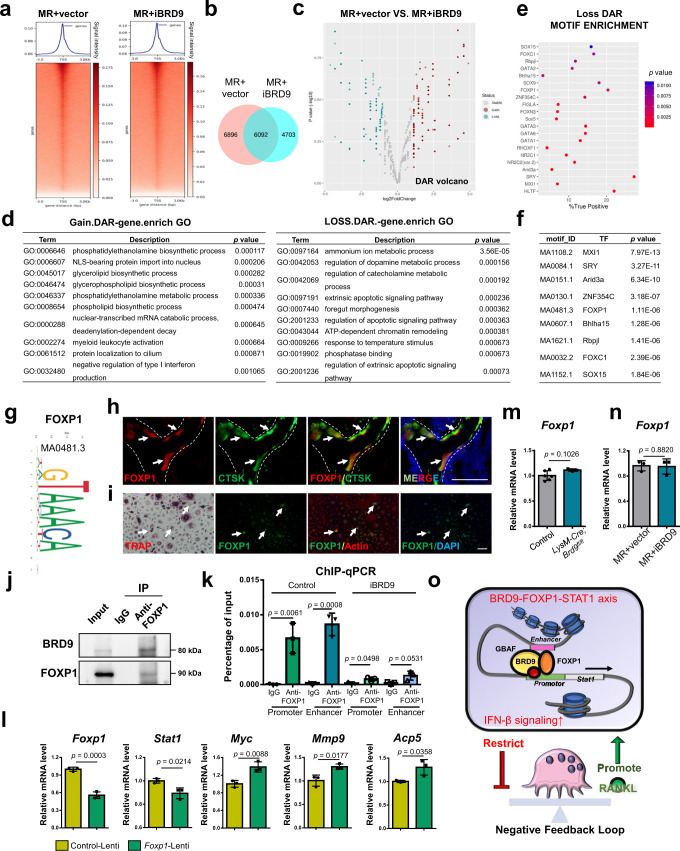
FOXP1 interacts with BRD9 to regulate *Stat1* transcription during osteoclastogenesis. **a** Heatmap showing the average ATAC-Seq signal centered on the transcription start site (TSS) of the nearest genes in osteoclastic induced BMDMs after control and iBRD9 treatment. Color scale represents average signal intensity. **b** Venn diagram showing the overlapping and discrepant peaks between control and iBRD9 group. **c** Volcano plot depicting differentially accessible region (DAR) between control and iBRD9 group. **d** GO terms of genes with gain and loss DAR around TSS after BRD9 inhibition. **e** Dot bubble plot showing the loss DAR motif enrichment after BRD9 inhibition. **f** Motifs enriched in BRD9 binding while loss after BRD9 inhibition. **g** The FOXP1 motif logo. **h** FOXP1 immunofluorescence (red) and CTSK (green) in the mouse distal femur at 4 weeks. Scale bar, 100 μm. **i** FOXP1 immunofluorescence (green), TRAP staining (red) and actin (red) in BMDMs after 3 days of RANKL-induction. Scale bar, 100 μm. **j** Co-IP assay with FOXP1 antibody (or IgG) in BMDMs during osteoclastic induction, followed by immunoblotting of BRD9 and FOXP1. **k** ChIP assay with FOXP1 antibody (or IgG) in RAW264.7 cell during RANKL-induction treated with iBRD9 or vector. *n* = 3 biologically independent samples. **l** The mRNA of *Foxp1*, *Stat1*, *Myc*, *Mmp9* and *Acp5* in osteoclastic induced RAW264.7 cell treated with *Foxp1* knockdown lentivirus (*Foxp1*-Lenti) or control lentivirus (Control-Lenti). *n* = 3 biologically independent samples. **m** The mRNA of *Foxp1* in BMDMs from *LysM-Cre;Brd9*^*fl/fl*^ (*n* = 3) and control mice (*n* = 6) at 4 weeks after 3 days of osteoclast differentiation. **n** The mRNA of *Foxp1* in BMDMs treated with iBRD9 after 3 days of osteoclast differentiation. *n* = 3 biologically independent samples. **o** Schematic drawing shows chromatin remodeling mediated by BRD9. All data in this figure are represented as mean ± SD. Negative binomial distribution used for **c**. Hypergeometric distribution test for **d**. the one-tailed Fisher’s Exact test for (**e**) and (**f**). Two-tailed Student’s *t*-test for (**m**), (**n**), (**k**), and (**l**). MR, M-CSF + RANKL. All experiments were performed in triplicates unless otherwise stated. Source data are provided in the Source data file.

The chromatin accessibility profile changed significantly with 12988 peaks in control group and 10795 peaks in BRD9 inhibition group, among which there are 6092 overlap peaks (Fig. 6a, b). Differentially accessible regions (DARs) between two groups were further compared and showed 3165 gain DARs and 2489 loss DARs after BRD9 inhibition (|log_2_(fold change)| > = 0.5) (Fig. 6c). Then the gain and loss DARs around TSS after BRD9 inhibition were annotated and enriched using GO analysis (Fig. 6d). Significant GO terms enriched in gain DAR-genes after BRD9 inhibition related to the phosphatidylethanolamine, glycerolipid, and phospholipid biosynthetic process, which were reported to promote osteoclast fusion^29,30^. And significant GO terms enriched in loss DAR-genes after BRD9 inhibition related to apoptotic signaling and catecholamine/dopamine metabolic process, which were reported suppressing osteoclast differentiation^31^. The change on the chromatin accessibility landscape after BRD9 inhibition suggests, beside of IFN-β signaling pathway, multiple affected processes could also contribute to BRD9-mediated negative regulation on osteoclastogenesis.

To identify potential interacting TFs of BRD9 during osteoclastogenesis, the top enriched loss motifs after BRD9 inhibition using the ATAC-seq data (Fig. 6e), were compared with BRD9 binding sites enriched motifs using the ChIP-seq data. Motifs enriched in BRD9 binding sites while loss after BRD9 inhibition suggests their potential cooperative interaction with BRD9, such as MXI1, SRY, Arid3a, ZNF354C, FOXP1 (Fig. 6f).

Among these motifs, we noticed FOXP1 (Fig. 6g), a member of the hepatic nuclear factor-3/forkhead domain family of winged-helix transcription factors, that is indispensable for normal development events^32^. Previous study has shown that overexpressing human *FOXP1* in monocyte/macrophage lineage cells using the CD68 promoter attenuated osteoclastogenesis and bone resorption activity in transgenic mice, suggesting the negative regulatory function of FOXP1 on osteoclast differentiation^33^. While the cellular mechanism and downstream signaling pathways is still unclear. We found that FOXP1 is expressed in CTSK + osteoclasts underneath the growth plate in the distal femur and osteoclasts (Fig. 6h, i). Based on the finding of its co-occupancy with BRD9 at genome above, we further hypothesized that FOXP1 may participate in the inhibitory function of BRD9 during osteoclastogenesis, at least partially through STAT1/IFN-β signaling pathway.

We scanned that FOXP1 TF-binding-site motif hits in the transcriptional regulatory region of *Stat1* and found there are multiple hits in the predicted proximal promoter region and enhancer region, where BRD9 binding (Supplementary Fig. 6). Then, co-immunoprecipitation (IP) assay confirmed the physical interaction between BRD9 and FOXP1 in BMDMs during osteoclast differentiation (Fig. 6j). To further validate the binding and transcriptional regulation function of FOXP1 on *Stat1*, we conducted ChIP-qPCR and *Foxp1* knockdown assay using lentivirus vector. As expected, we validated FOXP1 could bind to the *Stat1* promoter and enhancer directly, where BRD9 binding, while the binding function of FOXP1 at *Stat1* was compromised after BRD9 inhibition (Fig. 6k). And *Foxp1* knockdown with lentivirus vector leads to downregulated *Stat1*, accompanied with upregulation of osteoclastic differentiation genes compared with control lentivirus group, recapturing the regulatory function of BRD9 (Fig. 6l). We further excluded the possibility that it is the downregulated gene expression of *Foxp1* after BRD9 deletion or inhibition that leads to the compromised binding and regulatory function at *Stat1*, as shown with unchanged gene expression of *Foxp1* in BMDMs from *LysM-Cre;Brd9*^*fl/fl*^ mice compared with that from control littermates after RANKL-induction (Fig. 6m) and in BMDMs from wild-type mice after BRD9 inhibition (Fig. 6n).

In summary, these results clearly show that FOXP1 is one of the critical cofactors of BRD9 during *Stat1* transcriptional activation and their negative feedback regulation during osteoclastic differentiation (Fig. 6o).

### Function specificity of BRD9 distinguished from BRD4

**Fig. 7:**
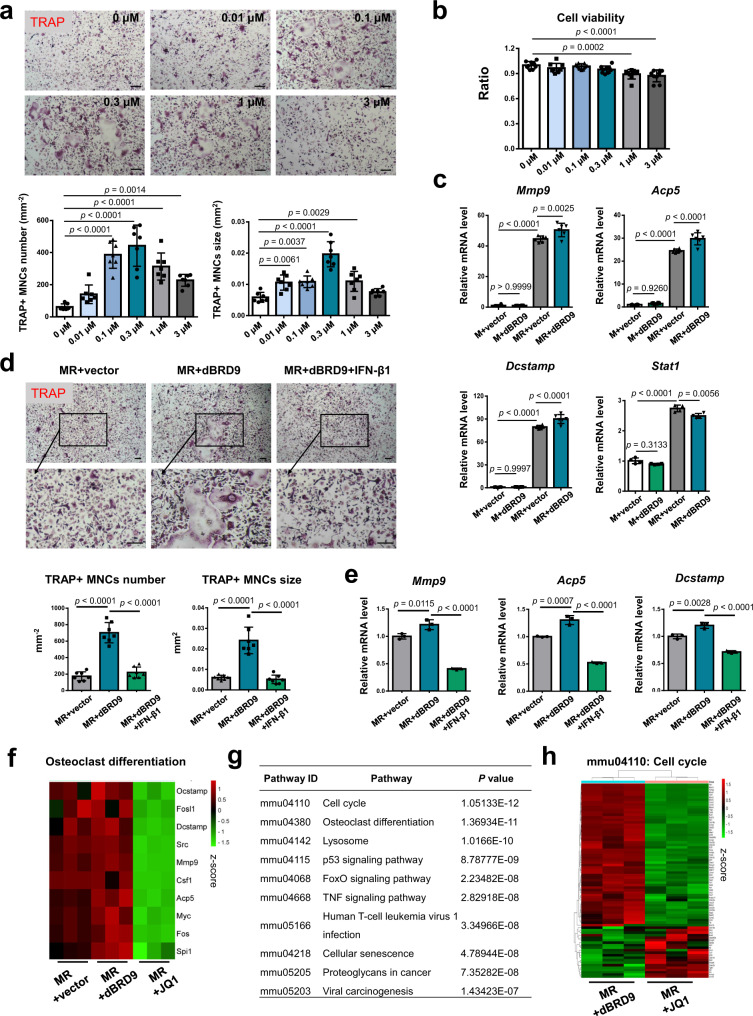
Function specificity of BRD9 distinguished from BRD4. **a** TRAP staining and quantification analysis of RANKL-induced BMDMs with 3 days of dBRD9 at different concentration. Scale bar, 200 μm. *n* = 7 biologically independent samples. **b** Cell viability of RANKL-induced BMDMs with 1 day of dBRD9 at different concentration, shown by cell counting kit 8 assay. *n* = 9 biologically independent samples. **c** The mRNA expression of *Mmp9, Acp5, Dcstamp,* and *Stat1* in BMDMs after 2 days of 0.3 μM dBRD9 or vector treatment with or without RANKL-induction, as measured by qPCR. *n* = 6 biologically independent samples for *Mmp9, Acp5, Dcstamp; n* = 4 biologically independent samples for *Stat1*. M-CSF, M; RANKL, R. **d** TRAP staining and quantification analysis of BMDMs treated with 3 days of 0.3 μM dBRD9 and 0.0625 ng/ml IFN-β1 during osteoclastic induction. Scale bar, 200 μm. *n* = 7 biologically independent samples. **e** The mRNA expression of *Mmp9, Acp5* and *Dcstamp* in BMDMs treated with 2 days of 0.3 μM dBRD9 and 0.0625 ng/ml IFN-β1 during osteoclastic induction, as measured by qPCR. *n* = 3 biologically independent samples. M-CSF, M; RANKL, R. **f** Heatmap showing the change on the transcriptional profile of critical osteoclast-related genes between dBRD9 and JQ1 treated BMDMs during osteoclastogenesis. **g** GO analysis shows the enriched top ten changed signaling pathway in JQ1-treated group compared with the dBRD9-treated group. **h** Heatmap showing the cell cycle-related signature genes in JQ1-treated group compared with the dBRD9-treated group during osteoclastogenesis. All data in this figure are represented as mean ± SD. Hypergeometric distribution test for **g**. One-way analysis of variance (ANOVA) with Dunnett’s multiple comparisons test for (**a**) and (**b**); with Tukey’s multiple comparisons test for (**c**), (**d**), and (**e**). Color scale in **f** and **h** represents normalized gene FPKM value by z-score scheme. All experiments were performed in triplicates unless otherwise stated. Source data are provided in the Source data file.

PROTACs (Proteolysis-Targeting Chimeras), the direct protein-specific degradation using bifunctional molecules with peptides to bridge E3 ligases and target protein ligand, are reported as a promising therapeutic strategy for various diseases in clinical trials^34^. We have further evaluated the function of BRD9 during osteoclastogenesis using a chemical PROTAC BRD9 degrader, dBRD9^35^. Consistently, we confirmed the dose-dependent increase on the number and size of TRAP + multinuclear differentiated osteoclasts from RANKL-induced BMDMs with dBRD9 treatment, without apparent cell toxicity under the concentration of 1 μM (Fig. 7a, b). The concentration of 0.3 μM dBRD9 was chosen for further evaluations for its most prominent function on promoting osteoclastogenesis. Consistent with the results using iBRD9, we identified IFN-β signaling activity, as one of the top downregulated signaling pathways after BRD9 degradation based on the transcriptional profile (Supplementary Table 1) and found that BRD9 degradation leads to enhanced expression of osteoclastogenic genes of *Mmp9*, *Acp5, Dcsstamp*, and downregulated expression of *Stat1* (Fig. 7c). Furthermore, the increased osteoclastogenesis after dBRD9 treatment was rescued after IFN-β signaling upregulation, as shown with the decreased number and size of TRAP + differentiated osteoclasts (Fig. 7d), as well as the downregulated expression of osteoclastogenic genes of *Mmp9*, *Acp5*, and *Dcstamp* (Fig. 7e).

It is reported that BRD9 regulates macrophage activation via cooperation with BRD4^12^. In contrast to our findings of the inhibition function of BRD9 on osteoclastogenesis, previous studies showed BRD4 inhibition suppressed RANKL-induced osteoclastogenesis^22^. To distinguish the function specificity of BRD9 with BRD4, we have compared the transcriptional profile after BRD9 degradation with dBRD9 and BRD4 inhibition with JQ1 during osteoclastogenesis. Besides of the apparent opposite change on the osteoclast-related genes (Fig. 7f), the enriched top ten changed signaling pathway in JQ1-treated group compared with the dBRD9-treated group were listed (Fig. 7g). We have noticed that the cell cycle and osteoclast differentiation process are on the top changed pathways in JQ1-treated group compared with dBRD9-treated group. The cell cycle-related signature genes were apparent downregulated in JQ1-treated group (Fig. 7h), suggesting their potential roles leading to the opposite effect between dBRD9 and JQ1 during osteoclastogenesis. The solid mechanism studies need to be fulfilled in the further.

### BRD9 degrader mitigates ZOL-related ONJ

ZOL-related ONJ is related to compromised osteoclastogenesis in patients after tooth extraction, with long-term medication of strong anti-bone resorptive agents^36^. In addition, recent studies have also unveiled that the pathogenesis of ONJ is also related to excessive macrophage activation and inflammation process^37^. While, no clinical procedure is available to prevent or effectively treat ONJ at present. Given the efficacy of chemical PROTAC dBRD9 shown in preclinical models of synovial sarcoma and leukemias^10,38^, we presumed that local treatment of dBRD9 degrader that characterized with both pro-osteoclastogenesis and anti-inflammatory response^12,13^, could prevent or mitigate ONJ effectively.

**Fig. 8:**
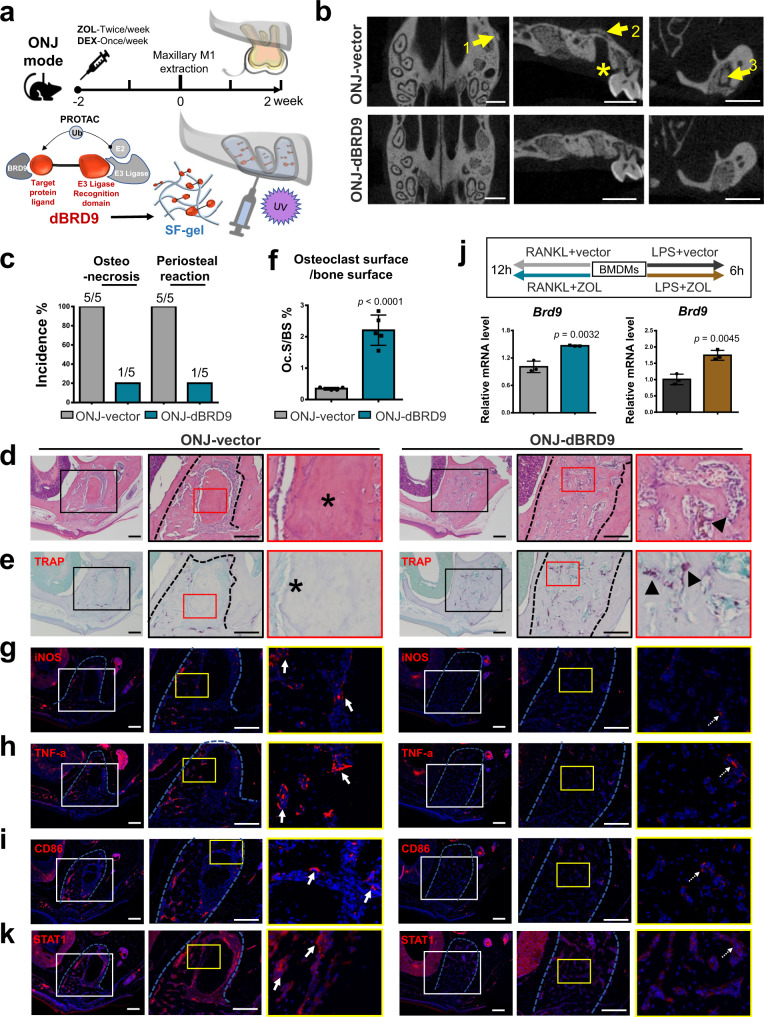
BRD9 degrader mitigates ZOL-related ONJ after tooth extraction. **a** Schematic illustrating the protocol used to establish ZOL-related ONJ model and dBRD9 degrader treatment. **b** μCT imaging of the maxillary bone from mice in dBRD9 degrader treated group and the control group. Yellow arrow 1 indicates severe periosteal reaction. Yellow arrow 2 indicates hyper-osseous maxillary sinus floor. Yellow arrow 3 indicates osteosclerotic patterns and sequestrum. Asterisk indicates insufficient amount of bone. Scale bar, 1 mm. **c** The incidence rate of osteonecrosis and periosteal reaction of the maxillary bone from mice in dBRD9 degrader treated group and the control group. *n* = 5. **d** H&E staining of the periodontal and alveolar bone area from mice in BRD9 degrader treated group and the control group. Colored boxes in the left are shown at higher magnification in right. Black dashed lines outline the boundary of the healed extraction sockets. Asterisk indicates necrotic alveolar bone with empty lacunae. Scale bar, 200 μm. **e** TRAP staining and **f** quantification of the periodontal and alveolar bone area from mice in BRD9 degrader treated group and the control group. Colored boxes in the left are shown at higher magnification in right. Arrows point to TRAP-positive osteoclasts. Asterisk indicates no signal. Scale bar, 200 μm. *n* = 5. Immunofluorescence staining of **g** iNOS (red), **h** TNF-a (red), **i** CD86 (red), **k** STAT1 (red) of the periodontal and alveolar bone area from mice in BRD9 degrader treated group and the control group. Colored boxes in the left are shown at higher magnification in right. Blue dashed lines outline the boundary of the healed extraction sockets. Arrows indicates positive signal. Scale bar, 200 μm. **j** The mRNA expression of *Brd9* in ZOL or vector-treated BMDMs during LPS-induced macrophage activation and RANKL-induced osteoclastogenesis at indicated timepoint. *n* = 3 biologically independent samples. All data in this figure are represented as mean ± SD. Two-tailed Student’s *t*-test for (**f**) and (**j**). All experiments were performed in triplicates unless otherwise stated. Source data are provided in the Source data file.

To test this hypothesis, we sought to develop a mouse model of ONJ-like disease by treating 6-week-old WT C57BL/6J mice with ZOL (125 μg/kg body weight; twice per week) and DEX (5 mg/kg body weight; weekly) for 4 weeks^39^. Two weeks after the initial ZOL/DEX injection, the maxillary left first molar was extracted from the mice. Silk fibroin (SF) has been widely used in bone tissue engineering due to its biocompatibility and controlled biodegradability^40^. In addition to providing suitable platforms for diverse chemical cues, the injectable and photocuring properties of SF entitled it as an ideal biomaterial for the bone regeneration of tooth extraction sockets in oral medicine. Therefore, a modified injectable photocuring SF containing BRD9 degrader was constructed and filled into the alveolar bone sockets immediately after tooth extraction, with SF containing vector as control group. After another two weeks, the maxillary bones in each group were collected for evaluation (Fig. 8a).

Alveolar bone healing assessment by μCT imaging showed severe periosteal reaction (Fig. 8b, arrow 1), hyper-osseous maxillary sinus floor (Fig. 8b, arrow 2) and insufficient amount of bone filled with osteosclerotic patterns and sequestrum (Fig. 8b, arrow 3) at the molar extraction site in the mice from ONJ-control vector group. While the molar extraction sockets in the mice from ONJ-dBRD9 group are filled with well-organized newly formed trabecular bone (Fig. 8b), with 80% reduced incidence rate of osteonecrosis and periosteal reaction compared within the mice from ONJ-control vector group (Fig. 8c). Histologic examination showed that the necrotic alveolar bone with empty lacunae in control mice (Fig. 8d, asterisk) were much attenuated in the healed extraction sockets in the dBRD9 group with well-organized bone tissue structure infilled with blood vessels (Fig. 8d, arrowhead).

TRAP staining and quantification analysis showed the locally increased osteoclasts that participating the active bone remodeling on the bone surface of the healed extraction sockets in dBRD9 degrader treated group comparing with the control group (Fig. 8e, f). Immunofluorescence staining of iNOS (Fig. 8g), TNF-α (Fig. 8h) and CD86 (Fig. 8i) showed the locally alleviated inflammatory response on the bone surface of the healed extraction sockets in dBRD9 degrader treated group comparing with the control group.

The well-matched indications of applying dBRD9 for ZOL-induced ONJ urger us to investigate the potential linkage between the pathogenesis and BRD9 function during both osteoclastogenesis and macrophage activation. Interestingly, our preliminary data showed that ZOL treatment could lead to upregulated expression of BRD9 in BMDMs during both RANKL-induced osteoclastogenesis and lipopolysaccharide (LPS)-induced macrophage activation (Fig. 8j). The finding recalls the function mechanism of BRD9, and also suggests the application of dBRD9 for ZOL-induced ONJ is one potential ideal etiotropic therapy. As expected, as one of the critical downstream targets of BRD9, STAT1 is downregulated in dBRD9 degrader treated group comparing with the control group in vivo (Fig. 8k).

Taken together, leveraging the advantages of pharmacological modulation of BRD9 and flexible injectable SF biomaterial scaffolds, we designed a local bone regeneration system for tooth extraction defects, which effectively prevents and mitigates ZOL-related ONJ. These results echo the molecular mechanism of BRD9 proved in our study, and more importantly provide a promising therapeutic strategy to prevent or effectively alleviate ONJ for patients with a long-term ZOL medication history in the future.

### BRD9 deletion/degradation mitigates LPS-induced bone resorption

It is well-documented that inflammatory stimuli usually upregulate RANKL expression in various types of cells such as bone marrow stromal cells and osteoblasts, and promote bone resorption, leading to overt bone loss in patients at clinical and animal models^41^. Given the inhibition role of BRD9 on anti-inflammatory response^12,13^, it is necessary to figure out the change on inflammation response and RANKL expression, besides of osteoclastogenesis, in the bone tissue of the BRD9-depleted mouse under both unstimulated and stimulated condition.

**Fig. 9:**
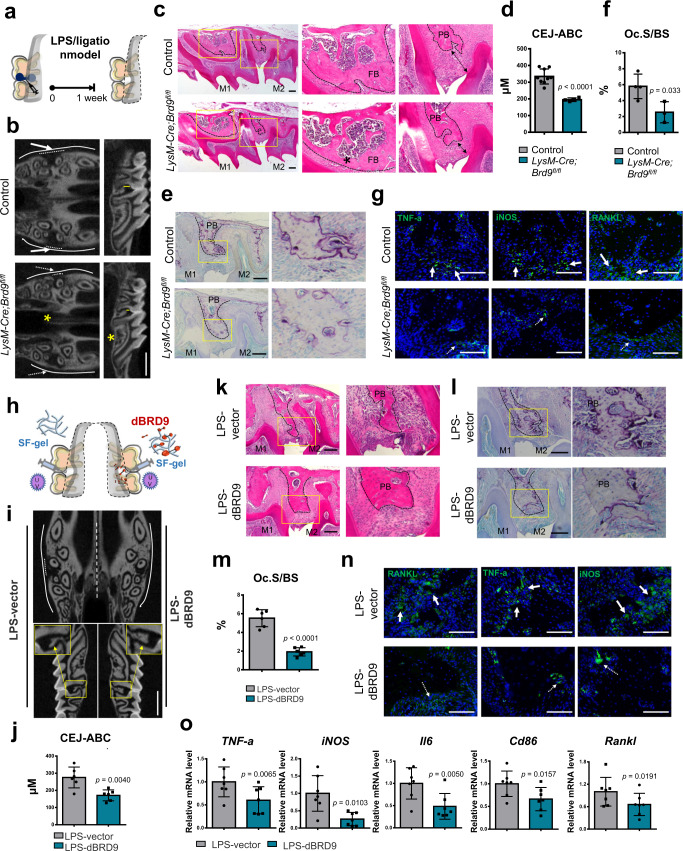
BRD9 deletion/degrader mitigates LPS-induced bone resorption. **a** Schematic illustrating the protocol for establishing LPS-induced localized aggressive periodontitis (LAP). **b** μCT imaging and **d** quantification of LPS-induced local alveolar bone loss in the maxillary bone from 4-week-old *LysM-Cre;Brd9*^*fl/fl*^ mice (*n* = 4) and littermate control mice (*n* = 9). Yellow line in **b** indicates cementum-enamel junction to alveolar bone crest (CEJ-ABC) distance (mm) in **d**. **c** H&E staining of LPS-stimulated maxillary bone from 4-week-old *LysM-Cre;Brd9*^*fl/fl*^ mice and littermate control mice. Arrows indicate distance between gingival sulcus bottom to alveolar bone crest. **e** TRAP staining and **f** quantification of LPS-stimulated maxillary bone from 4-week-old *LysM-Cre;Brd9*^*fl/fl*^ mice (*n* = 3) and littermate control mice (*n* = 4). **g** TNF-a, iNOS and RANKL immunofluorescence (green) in LPS-stimulated maxillary bone from 4-week-old *LysM-Cre;Brd9*^*fl/fl*^ mice and littermate control mice. **h** Schematic illustrating dBRD9 degrader treatment. **i** μCT imaging and **j** quantification of LPS-induced local alveolar bone loss in BRD9 degrader treated (right) and control (left) lateral of the maxillary bone from 6-week-old mice. *n* = 6. **k** H&E staining, **l** TRAP staining, and **m** quantification of LPS-induced local alveolar bone in BRD9 degrader treated and control lateral of the maxillary bone from 6-week-old mice. *n* = 6. **n** RANKL, TNF-a, and iNOS immunofluorescence (green) in LPS-induced local alveolar bone in BRD9 degrader treated and control lateral of the maxillary bone from 6-week-old mice. **o** The mRNA expression of *TNF-a*, *iNOS*, *Il6*, *Cd86,* and *Rankl* of LPS-induced local alveolar bone in BRD9 degrader treated and control lateral of the maxillary bone from 6-week-old mice. *n* = 7. All data in this figure are represented as mean ± SD. Two-tailed Student’s *t*-test for (**d**), (**f**), (**j**), (**m**), and (**o**). M1, first molar. M2, second molar. Asterisk in **b**, **c** indicates less bone mass. Scale bar in **b**, **i**, 1 mm; **c**, **e**, **k**, **l**, 200 μm; **g**, **n**, 100 μm. All experiments were performed in triplicates unless otherwise stated. Source data are provided in the Source data file.

The results showed that, without additional infection, loss of *Brd9* led to slight inhibition on the basal expression of proinflammatory cytokines, such as TNF-α, iNOS, CD86, IL-1b with no apparent effect on RANKL in the femur bone tissue as shown with immunofluorescence staining, as well as the quantification analysis using qPCR (Supplementary Fig. 7). Localized aggressive periodontitis (LAP) is a common disease in oral medicine, which is characterized with hyper-inflammatory response and acute severe bone loss. Therefore, LPS-induced in periodontal alveolar bone loss was used as a model to evaluated the change on inflammation response and RANKL expression, besides of osteoclast activity, in the bone tissue of the *Brd9*-depleted mouse (Fig. 9a). Interestingly, although carrying osteoporosis phenotype (Fig. 9b, c, asterisks), the *Brd9*-depleted mouse showed resistant to LPS/ligation-stimulated local acute damage on the periodontal alveolar bone (Fig. 9b–d) and osteoclast activity (Fig. 9e, f) compared with the control mice. Cellular and molecular examination results suggested the alleviated acute local bone loss and osteoclast activity might be benefit from the resistance to LPS/ligation-stimulated hyper-inflammatory response and increased RNAKL in *LysM-Cre;Brd9*^*fl/fl*^ mice compared with control mice, as shown with immunofluorescence staining of TNF-α, iNOS, and RANKL (Fig. 9g).

Inspired by above findings, to test the prevention and therapeutic potential of dBRD9 for LPS/ligation-induced acute periodontitis and alveolar bone loss, the modified injectable photocuring SF containing BRD9 degrader, was further utilized and injected into the periodontal tissue during LPS/ligation induction, with SF containing vector in the contralateral (Fig. 9h). After 5 days, the alveolar bones in each group were collected for evaluation. The results showed that LPS/ligation-stimulated acute local bone loss (Fig. 9i–k), osteoclast activity (Fig. 9l, m), as well as macrophages activation and RANKL expression (Fig. 9n, o) in the periodontal alveolar bone were much attenuated in the dBRD9 group compared with control vector group.

Taken together, the anti-inflammatory effect of BRD9 deletion or degradation in bone tissue is likely more prominent under infection stimulated circumstance compared within the basal condition.

## Discussion

With the deepening understanding on the epigenetics during key developmental processes in organogenesis, an increasing number of studies have defined epigenetic changes as determinants of gene expression in osteoclastogenesis^42–44^. For example, the demethylation of H3K27me3 in the *Nfatc1* gene locus by Jumonji domain-containing 3 plays a critical role in osteoclast differentiation^42^; KDM4B-CCAR1-MED1 signaling axis induces euchromatinization changes near the promoters of osteoclast-related genes through H3K9 demethylation^44^. While the relationship between chromatin remodeler and osteoclast differentiation remains incompletely defined. Recent studies revealed that BRD9-mediated chromatin remodeling cooperates with TFs to modulate the expression of lineage-specific or stimulus-induced genes in cancer cell proliferation^45^, pluripotency^9^, inflammatory responses^12,13,15^ and antiviral activity^46,47^. For example, in mouse embryonic stem cells, BRD9 targets distinct genomic features from those targeted by canonical BAF complex and maintains members of the transcriptional network associated with pluripotency^9^; in cytokine stressed β-cells, inhibition of BRD9 increases the anti-inflammatory function of vitamin D receptor (VDR) through modulating VDR and BRD9 interaction to limit β-cell failure^15^; in macrophages, BRD9 acts as a genomic antagonist of glucocorticoid receptor (GR) at inflammatory-related genes, restricting the therapeutic efficacies of glucocorticoids on repressing inflammatory responses^13^. While the role of BRD9 in osteoclast formation is only now being addressed in our study. We revealed that BRD9 interacts with FOXP1 and provides chromatin accessibility for it at *Stat1* promoter and enhancer, activating IFN-β signaling thereafter. The finding of transcriptional activator role of BRD9 echoes the result from previous study that BRD9-binding sites are mainly associated with active chromatin regions in genome^7^, and also highlights the context-dependent roles of BRD9-containing ncBAF complex and its diverse cofactors in maintaining transcriptional networks in physiological and pathological process. Nevertheless, BRD9 is a rather conserved positive regulator of the Type I interferon signaling pathway and interferon-stimulated genes in multiple cell types whether that function is inflammatory^12,13^, antiviral^46,47^, or negative feedback as in the case of osteoclastogenesis in our study.

Previous mass spectrometry-based interaction proteomics experiments identified bromodomain as a major acetyl-lysine readers, associating with regulatory active enhancers^15,48^. IBRD9 is BRD9 bromodomain inhibitor and functions via impairing the acetyl-lysine recognition of BRD9^14^. Besides of proximal promoter, we observed enhancer-associated transcriptional abnormalities at *Stat1* in RAW264.7 cells after BRD9 inhibition, indicating that the regulatory role of BRD9-containing ncBAF, at least in part, via bromodomain-mediated interactions with chromatin regions marked with acetyl-lysine modifications.

Similar with BRD9, the bromodomain and BET protein family (BRD2, BRD3, BRD4, and BRDT) is also an important subfamily of bromodomain protein superfamily^49^. Interestingly, previous work demonstrated that a pan-BET protein inhibitor I-BET151 or a typical class of BRD4 inhibitors JQ1, suppressed osteoclast formation with inhibition on TRAP, MMP-9, and CTSK, by blocking RANKL-mediated MYC-NFAT axis, MAPK, and NF-κB pathways^50–53^. Therefore, although a recent study declaims that BRD9 regulates macrophage activation via cooperation with BRD4^12^, it is not likely the same case as for osteoclastogenesis. Our preliminary data showed the downregulated genes in JQ1-treated group compared with dBRD9-treated group, are enriched on cell cycle, ribosome biogenesis and DNA replication. Actually, BRD4 is reported conducting critical and general role in transcription regulation, in the genome of various normal and transformed cell types^54,55^. As such, *Myc*, which is essential for both cell cycle in tumorigenesis and TF regulatory network in osteoclastogenesis, is one conserved target of BRD4^22,52,56^. Therefore, BRD4-mediated transcription activation of genes, such as *Myc*, could entitle BRD4 the tumorigenic role and also positive role in osteoclastogenesis, which is distinct with the function of BRD9 demonstrated in our study. Solid mechanism studies with high-resolution insights into these transcriptional regulation processes mediated by these distinct factors needs to be explored in further studies.

FOXP1 contains both DNA-binding- and protein-protein binding-domains. It is reported that FOXP1-mediated repression of Jagged1 is pivotal in regulating embryonic neural stem cells differentiation^57^; FOXP1 binds to CSF1R promoter with corepressor NCOR2 involving in regulation of monocyte differentiation and macrophage functions^33^; and FOXP1 physically interacts with silencing mediator for retinoid and thyroid receptor and recruited to the *p21* promoter during myocardial development^58^. In bone tissue, it is also demonstrated that FOXP1 transcriptionally controls senescence and fate commitment of BMSCs via directly represses transcription of p16INK4A at promoter region or repress adipogenesis and osteogenesis-associated genes through interactions with the CEBPβ/δ complex or RBPjκ, respectively^59^. Contrary to its well-known repressor or corepressor role during organogenesis, our study has presented that FOXP1 binds to previously uncharacterized sites within the proximal promoter and enhancer of *Stat1*, functioning as a transcriptional co-activator with BRD9. This finding of the cooperation between FOXP1 and chromatin remodeler BRD9 not only expands its pleiotropic gene regulatory function, but also further highlights its cell type-specific manner during organogenesis.

Beside of FOXP1, other TFs could also cooperate with BRD9 during the negative feedback regulation during osteoclastogenesis. For example, it is reported that MXI1 (Max interactor 1), interacts specifically with Max to form heterodimers antagonizing the transcription activation of MYC^60^. Although there is lack of direct function evidence, the critical role of MYC in osteoclast differentiation implicates the potential repressor role of MXI1 during osteoclastogenesis. In addition, the decreased SRY (sex-determining region Y) is reported to related with the increased incidence of osteoporosis in human bone samples^61^. The potential cooperative interaction of BRD9 with these enriched TFs, further emphasized the critical role of BRD9-mediated chromatin remodeling during the negative regulation on osteoclastogenesis.

In this study, we confirmed the synergizing function of BRD9 inhibition and glucocorticoids during osteoclastogenesis, and the functional categories of iBRD9/DEX cooperatively regulated genes were enriched on metabolic process during osteoclastogenesis. Although it is distinct with iBRD9-alone regulated gene network, we still could get inspires from Wang et al.’s study^13^. The authors found that during macrophage activation, BRD9 loss could affect the glucocorticoid response of a set of GR binding sites that associated with cell metabolism and cell–cell adhesion, although without significant colocalization of BRD9, while through redistributing their GR binding to the sites associated with the inflammatory response gene network. While the underling sophisticated interaction and regulation network needs to be explored in further studies.

Our preliminary data showed that ZOL treatment leads to upregulated expression of BRD9 in BMDMs during both osteoclastogenesis and macrophage activation. The finding recalls the function of BRD9 on restricting osteoclastogenesis and promoting inflammatory response^12,13^, and also suggests the application of dBRD9 for ZOL-induced ONJ is one ideal etiotropic therapy. It provides clues extending our understanding on bisphosphonates mechanism of action and is worth further investigation. In addition, we also find that the acute bone loss caused by LPS-induced hyper-inflammatory response is kind of exempted from BRD9 deletion/degrader during the pathogenesis. Therefore, the complex osteoimmunology underlying the function of BRD9 on osteoclastogenesis and inflammatory response in physiological and pathological condition, is remained to be investigated deeply.

In summary, our study has identified a BRD9-FOXP1-STAT1 axis that is indispensable for the negative feedback loop and homeostasis of osteoclasts. These findings further expand our knowledge of the delicate interaction network among chromatin remodelers, TFs, and signaling pathways the homeostasis of bone-immune circuit, and also shed light on developing attractive therapeutic strategies for osteoclast-associated bone disease in the future.

## Methods

### Mice

*Brd9-flox* mice (Strain NO. T008489), *LysM-Cre* mice (Strain NO. T003822), and WT C57BL/6J mice (Strain NO. N000013) from GemPharmatech (Nanjing, China) and tdTomato (Strain NO. 007909) mouse line^62^ from Jackson Laboratory (Maine, USA) were used and cross-bred in this study. All mice were used for analysis regardless of sex. All mice were housed in pathogen-free conditions with constant ambient temperature (22 ± 2 °C) and humidity (55 ± 10%), with an alternating 12-h light/dark cycle. All mice were euthanized by carbon dioxide overdose followed by cervical dislocation. All animal studies were approved by the Institutional Animal Care and Use Committee at Ninth People’s Hospital, School of Medicine, Shanghai Jiao Tong University (SH9H-2022-A926-1).

### ZOL-related ONJ model

Six-week-old WT C57BL/6J mice were subjected to an injection of 125 μg/kg body weight ZOL (446, S1314, Selleck) intraperitoneally twice a week and 5 mg/kg body weight DEX (Sigma-Aldrich, D-085) subcutaneously weekly for 4 weeks (*n* = 5 per group). Under general anesthesia, the left maxillary first molar was atraumatically extracted 2 weeks after the initial injection. Silk fibroin protein was extracted from Bombyx mori cocoons^40^. Briefly, de-sericin process was performed by boiling Bombyx mori cocoons in Na_2_CO_3_ solution. After dissolved and dialyzed, the regenerated silk solution (3% w/v) was further frozen before lyophilization. To improve its stability in aqueous environments, scaffolds were autoclaved to induce β-sheet formation. Then dBRD9 (R&D, 6606) was mixed within the silk fibroin hydrogel at the final concentration of 0.3 mM before injection. Two weeks after the tooth extraction, the mice were sacrificed to harvest the maxilla from each mouse for μCT imaging, histologic and molecular examination.

### LPS/ligation induced periodontal bone loss model

To evaluate LPS-induced in periodontal alveolar bone loss, the 5-0 silk sutures soaked in 2 mg/ml LPS (Sigma-Aldrich, L2880) were placed between the cervical of the first and second maxillary molars in control and *Brd9*-depleted mouse under general anesthesia at 4-week-old. One week after the LPS/ligature application, the mice were sacrificed to harvest the maxilla from each mouse and fixed. After μCT scanning, these tissues were decalcified before processed for histological analysis. To test the prevention and therapeutic potential of dBRD9 for LPS-induced alveolar bone loss, the modified injectable photocuring SF containing BRD9 degrader, mentioned above, was further utilized and injected into the periodontal tissue during LPS/ligation induction in six-week-old WT C57BL/6J mice, with SF containing vector in the contralateral. After 5 days, the alveolar bones in each group were collected for μCT imaging, histologic and molecular examination.

### Micro-CT analysis

Micro-CT (μCT) analysis of fixed femur or maxillary bone was performed using a Skyscan 1176 (Bruker, Kontich, Belgium) at 50 kVp, 450 μA and a resolution of 9 μm. The obtained images were reconstructed with NRecon software (v1.7.1.0, Bruker, Kontich, Belgium). The region from 50 to 250 slices below the growth plate was analyzed for BV/TV, Tb.Th., Tb.N., and Tb.Sp, and the region from 500 to 550 slices below the growth plate was analyzed for Ct.Th and Ct.Pm using the program CTAn (v 1.16, Bruker, Kontich, Belgium).

### Histological analysis

Mouse femur or maxillary bone were dissected and fixed, followed by decalcification in 10% EDTA in PBS for 1–3 weeks depending on the age of the samples. The decalcified samples were dehydrated with serial ethanol and xylene and embedded in paraffin. The paraffin-embedded samples were then cut into sections with a thickness of 4 mm using a microtome (Leica). H&E staining (servicebio, G1005), von kossa staining (servicebio, G1043), TRAP Staining (sigma, 387 A) and ALP staining (Beyotime, C3206) were conducted following the manufacturer’s standard protocol. For dynamic bone histomorphometric analysis, double fluorescence labeling was performed^63^. Briefly, calcein (Sigma-Aldrich, C0875, 25 mg/kg) and Alizarin Red S (Sigma-Aldrich, A5533, 30 mg/kg) were administered to mice by intraperitoneal injection at 7 d and 2 d before sacrifice, respectively. Then the uncalcified bone tissues were dehydrated and embedded before sectioned at 8 mm. Nikon Eclipse Ti-U Microscope (Nikon, Japan) with NIS-Elements software (v4.5000.1117.0) and Zeiss Axio Scope A1 Microscope (ZEISS, German) with DP2-TWAIN software (v3.0.0.6212) were used to acquire images.

### Immunofluorescence staining

The decalcified samples were dehydrated in serial sucrose solutions and embedded in OCT compound (Tissue-Tek, Sakura). OCT-embedded samples were cryosectioned at 8 mm using a cryostat (Leica CM1850) followed by staining. For immunofluorescence staining, cryosections were soaked in blocking solution for one hour at room temperature and then incubated with primary antibodies diluted in blocking solution at 4 °C overnight. After washing three times in PBS, the sections were incubated with alexa-conjugated secondary antibodies and counterstained with DAPI. Antibodies used in immunofluorescence staining as following: BRD9 antibody (Abcam, ab259839, 1:100), CTSK antibody (Abcam, ab37259, 1:100), STAT1 antibody (Cell signaling, 9172, 1:100), FOXP1 antibody (Merck Millipore, ABE68, 1:100), RUNX2 antibody (Thermo Fisher Scientific, MA5-41185, 1:100), iNOS (Abcam, ab178945, 1:100) TNF-a (Thermo Fisher Scientific, PA5-19810, 1:100), CD86 (Cell signaling, 19589, 1:100), RANKL antibody (bioworlde, BS72037, 1:100), Goat anti-Rabbit IgG (H + L) Cross-Adsorbed Secondary Antibody, Alexa Fluor 488 (Thermo Fisher Scientific, A-11008, 1:200), Donkey anti-Rabbit IgG (H + L) Highly Cross-Adsorbed Secondary Antibody, Alexa Fluor 594 (Thermo Fisher Scientific, A-21207, 1:200), Goat anti-Mouse IgG (H + L) Cross-Adsorbed Secondary Antibody, Alexa Fluor 488 (Thermo Fisher Scientific, A-11001, 1:200) and Goat anti-Mouse IgG (H + L) Cross-Adsorbed Secondary Antibody, Alexa Fluor 594 (Thermo Fisher Scientific, A-11005, 1:200).

### Cell culture

Bone marrow cells were harvested from murine femurs of four to six-week-old WT C57BL/6J mice and cultured in α-minimum essential medium (α-MEM) containing 10% FBS and 1% penicillin-streptomycin overnight (Thermo Fisher Scientific, Waltham, MA, USA). For osteoclast induction, BMDMs were cultured into complete media with 50 ng/ml recombinant soluble murine M-CSF (PeproTech, 315-02) and 100 ng/ml recombinant soluble murine RANKL (PeproTech, 315-11). For activation of macrophages, BMDMs were cultured into complete media supplemented with 100 ng/ml LPS. RAW264.7 cells (Cyagen Biosciences, M3-0101) were maintained in DMEM media containing 10% FBS, 1% penicillin/streptomycin^63^. For osteoclast induction, RAW264.7 cell were cultured in presence of 100 ng/ml RANKL.

For the BRD9 inhibitor/degrader experiments, the cells were treated with either vehicle or varying concentrations of iBRD9 (Tocris Bioscience, 5591) or dBRD9 (R&D Systems, 6606) twenty-four hours after plated, as well as IFN-β1 cytokine (0.0625 ng/ml, R&D Systems, 8234-MB), DEX (10^−11^−10^−9 ^M), JQ1 (0.25 μM, Abmole Bioscience, M2167) and ZOL (10 μM, Abmole Bioscience, M5032). The cell viability was evaluated using Cell Counting Kit-8 (Cck8, CK04, Dojindo, Japan) after incubation for another twenty-four hours. For all the cultures, media was changed every two days until indicated timepoints.

### Lentivirus and plasmids transfection

*Stat1*-overexpression lentivirus (*HBLV-m-Stat1-HIS-ZsGreen-PURO*) with its control lentivirus (*HBLV-ZsGreen-PURO*), and *Foxp1*-knockdown lentivirus (*HBLV-m-Foxp1 shRNA1-NEO*) with its control lentivirus (*HBLV-NEO NC*) were purchased from Hanbio Biotechnology Co. Ltd (Shanghai). For lentivirus transfection, 2 × 10^5^ Raw264.7 cells were seeded in 12-well plates in α-MEM overnight and were infected with lentivirus expressing either target gene or control lentivirus at a multiplicity of 20. After 24 h, transfected Raw264.7 cell line were induced with 100 ng/ml RANKL for further test.

### Quantitative reverse transcription PCR (qPCR)

For qPCR analysis, RNA was extracted using TRIzol (Sigma, T9424) and was reverse-transcribed with the Prime Script RT master kit (TakaRa Bio Inc., RR036A). Then the relative amounts of each mRNA transcript were analyzed using Roche LightCycler 480 system (v1.5.1.74) with SsoAdvanced Universal SYBR Green Supermix (Bio-Rad, 1725270). The expression of β-actin as an internal control. Primer sequences are listed in Supplementary Table 2.

### RNA-sequencing analysis

For RNA-sequencing analysis, libraries were prepared using NEBNext Ultra II RNA Library Prep Kit and then sequenced on Illumina NovaSeq 6000 platform. Raw reads were filtered using Cutadapt (v1.15) and aligned with the GRCm39 genome using HISAT2 v2.0.5. Read Count values on each gene were compared using HTSeq (v0.9.1) and normalized to FPKM. Then difference expression of genes was analyzed using DESeq (v1.30.0) (fold change > = 1.2 and *p* < 0.05) and R language Pheatmap (v1.0.8) software package. GO enrichment analysis of the different expressed genes was performed using topGO (v2.40.0) (*p* < 0.05). The enrichment analysis of the KEGG pathway of differential genes was performed using ClusterProfiler (v3.4.4) software (*p* < 0.05). GSEA analysis was performed to functionally annotate the relevant genes and assess the enriched signaling pathways using GSEA_Linux_4.1.0.

### Western blot and co-immunoprecipitation (co-IP)

For western blot analysis, total protein was obtained and homogenized in RIPA buffer (Cell Signaling, 9806 s) supplemented with protease inhibitor (Thermo Fisher Scientific, A32959). Western blot was performed per standard protocol and signals were detected using UVITEC Alliance system (v16.0.3.0). Uncropped and unprocessed scans of the blots are provided in the Source Data file. Antibodies used in western blot as follows: BRD9 antibody (Abcam, ab259839, 1:1000), MMP9 antibody (Abcam, ab228402, 1:1000), CTSK antibody (Abcam, ab37259, 1:1000), ACP5 antibody (Abcam, ab235448, 1:1000), β-actin antibody (Abcam, ab20272HRP, 1:5000), FOS antibody (Cell signaling, 4384, 1:1000), IFN-β1 antibody (Cell signaling, 97450, 1:1000), STAT2 antibody (Cell signaling, 72604, 1:1000), STAT1 antibody (Cell signaling, 9172, 1:1000), FOXP1 antibody (Cell signaling, 4402, 1:1000), Mouse IgG HRP-conjugated antibody (R&D, HAF007, 1:1000) and Rabbit IgG HRP-conjugated antibody (R&D, HAF008, 1:1000).

For co-IP, BMDMs after osteoclast induction were harvested and lysed in Pierce™ IP Lysis Buffer (Thermo Fisher Scientific, 87787). Then lysates were subjected to immunoprecipitation with anti-FOXP1 antibody (Cell signaling, 4402, 1:200) or normal Rabbit IgG (Cell Signaling, 2729, 1:4000) and protein A-Sepharose (VWR, CA97067-898). Immune complexes were washed and subjected to immunoblotting with anti-FOXP1 (Cell Signaling, 4402, 1:1000) or anti-BRD9 (Abcam, ab259839, 1:1000) antibodies.

### Dual luciferase reporter assays

The predicted *Stat1* proximal promoter region (2.6 kb, −2995 ~ +576) and enhancer region (1.2 kb, −6338 ~ −5138) was synthesized and cloned into pGL3-basic luciferase reporter as and pGL3-basic-2.6 kb (promoter) and pGL3-basic-3.8 kb (promoter + enhancer). For transfection, 2 × 10^5^ Raw264.7 cell line were seeded in 12-well plates in α-MEM overnight and were infected with above reporter clones using Lipofectamine 3000 transfection reagent (Thermo Fisher Scientific, L3000008) and Opti-MEM™ I Reduced Serum Medium (Thermo Fisher Scientific, 31985062) according to the manufacturer’s protocol. After 24 h incubation, transfected Raw264.7 cell line were treated with iBRD9 or control vector cultured in presence of 100 ng/ml RANKL. After another 24 h incubation, the cell was lysed for dual luciferase reporter gene assay kit (Beyotime, RG027) according to the manufacturer’s instructions. Renilla luciferase is used as an internal standard control, and the normalized reporter activity of all samples was compared. Each group had five replicate samples. Independent experiments were repeated in triplicate.

### ATAC-sequencing analysis and motif analysis

5000 cells from each group of osteoclastic induced BMDMs treated with iBRD9 or control vector for 24 h were used for preparation of ATAC-seq libraries according to the protocol described by Kaestner Lab (https://www.med.upenn.edu/kaestnerlab/protocols.html). The transposed DNA libraries were sequenced on Illumina Novaseq 6000 system with PE150 mode. Library sequencing quality was assessed using Cutadapt (v1.9.1). Trimmed libraries were aligned to the GRCm39 mouse genome using Bowtie 2 (v2.2.6)^64^. PCR duplicates were removed and the number of mapped reads downsampled to a standard of ~20 M using Picard (v1.126) (http://broadinstitute.github.io/picard/). ATAC-Seq peaks were called subsequently using MACS (v3.0.0a6)^65^ (-f BAMPE -B --SPMR –keep-dup all). Then the high-confidence set of peaks from all samples were merged to acquire consensus peakset. Potential DARs from consensus peakset were annotated with |log_2_(fold change)| >= 0.5. Motif analysis on DARs was performed by MEME (v5.4.1) suite with default settings with *p* < 0.01^66^. Gene ontology enrichment was performed using R package ClusterProfiler (v4.6.0), with default settings.

### ChIP assay and integrative analysis

BMDMs were osteoclastic induced in presence of 50 ng/ml M-CSF and 100 ng/ml RANKL for 3 days and fixed with formaldehyde after amplification for further ChIP-sequencing analysis. RAW264.7 cell were osteoclastic induced in presence of 100 ng/ml RANKL for 3 days and fixed with formaldehyde after amplification for further ChIP-qPCR analysis. The Simple ChIP Plus Enzymatic Chromatin IP Kit (Magnetic Beads; Cell Signaling Technology 9005), anti-FOXP1 antibody (Cell signaling, 4402, 1:100) with normal Rabbit IgG (Cell Signaling, 2729, 1:2000) as a non-specific IgG control, anti-BRD9 antibody (Bethyl Laboratories, A700-153, 1:100) with normal Rabbit IgG (Cell Signaling, 2729, 1:100) as a non-specific IgG control were used following the manufacturer’s instructions. The *Stat1* ChIP primers for binding site 1 (BS1, −40 ~ +52) and binding site 2 (BS2, −5566 ~ −5424) amplified a region at the promoter and enhancer, respectively. Primer sequences are listed as follows. Stat1-BS1-F: AACAGCCGGCCAATCTCTG; Stat1-BS1-R: GAAAACCGAAAGTACCGGGC; Stat1-BS2-F: CCGGCTGAGTTCCCAGAAAG; Stat1-BS2-R: AGGCTTTGCTTTAGGACCCC.

ChIP-sequencing libraries were prepared using the KAPA HTP Library Preparation Kit and were sequenced on Illumina Novaseq 6000 system. Double-end sequencing was used with 150 bp read length. Raw reads were filtered using FASTX-Toolkit (v0.0.14) (http://hannonlab.cshl.edu/fastx_toolkit/). Then the clean reads were aligned to the GRCm39 mouse genome using Bowtie 2 (v2.3.5.1)^64^. Multiple aligned reads were filtered out using Picard (v2.27.5) (http://broadinstitute.github.io/picard/). Then the unique mapped reads without duplicated reads were called for peaks using MACS2 (v2.2.71)^65^ with default parameters and *p* < 0.05. The peaks were annotated using ChIPseeker (v1.20.0)^67^ and motif analyses were carried out using the MEME (v4.1.2)^66^.

For integrative analysis of RNA-Seq with ChIP-Seq, venn analysis was initially performed between the overlapping of RANKL-induced and iBRD9 downregulated genes (|log_2_(fold change)| > = 0.1 and *p* < 0.05) using the RNA-sequencing datasets. And the overlapping genes were further compared with the BRD9 binding profiles during osteoclastogenesis using Venn analysis. Then, KEGG enrichment and PPI analysis were conducted with the overlapping 717 genes where BRD9 binding, RANKL-induced while downregulated after BRD9 inhibition. To identify potential interacting TFs of BRD9 during osteoclastogenesis, the top enriched loss motifs after BRD9 inhibition using the ATAC-seq data and the BRD9 binding sites enriched motifs using the ChIP-seq data were further integrative analyzed.

### Statistical and reproducibility

All statistical analyses were performed with GraphPad Prism v6.01 software and are presented as mean ± standard deviation. Unpaired two-tailed t-test between two groups or one-way analysis of variance (ANOVA) with Tukey’s or Dunnett’s multiple comparisons post hoc test among three or more groups was used for comparisons. *P* < 0.05 is considered statistically significant. All experiments were repeated in triplicate or more unless otherwise stated.

### Reporting summary

Further information on research design is available in the Nature Portfolio Reporting Summary linked to this article.

## Supplementary information

Supplementary Information

Reporting Summary

## Data Availability

The mRNA, ATAC, and ChIP-sequencing data generated in this study have been deposited in the Gene Expression Omnibus database under accession code GSE222240. GRCm39 genome is referenced in this study [http://asia.ensembl.org/Mus_musculus/Info/Index]. The other relevant data generated in this study are provided in the Supplementary Information/Source data file. Source data are provided with this paper.

## Source data

Source Data
