# Supplementary material for: BRD9-mediated chromatin remodeling suppresses osteoclastogenesis through negative feedback mechanism
Source: Nat Commun. 2023 Mar 14;14:1413. doi: 10.1038/s41467-023-37116-5 (PMC10014883; doi:10.1038/s41467-023-37116-5)
Supplement: Supplementary file 1 — Supplementary Information [file 41467_2023_37116_MOESM1_ESM.pdf]

BRD9-mediated chromatin remodeling suppresses osteoclastogenesis through negative feedback mechanism

Supplementary Information

Supplementary Figures

Supplementary Fig. 1 *Brd9* knockout strategy and efficiency in *LysM-Cre;Brd9$^{fl/fl}$* mice.

Supplementary Fig. 2 Directed acyclic graph of the top 10 GO terms for biological process in the comparison between BMDMs in MR+vector and MR+iBRD9 group.

Supplementary Fig. 3 Synergizing function of BRD9 inhibition with glucocorticoid during osteoclastogenesis.

Supplementary Fig. 4 Genomic location of the region cloned in *Stat1* luciferase reporter plasmids and primer sets used in ChIP assay.

Supplementary Fig. 5 Genomic location of the SNPs in predicted transcriptional regulatory region of *Stat1*.

Supplementary Fig. 6 FOXP1 motif hits in the predicted transcriptional regulatory region of *Stat1.*

Supplementary Fig. 7 loss of *Brd9* led to slight inhibition on the basal expression of proinflammatory cytokines in the femur bone tissue.

Supplementary Table

Supplementary Table 1: The top 10 GO biological process terms enriched in downregulated genes in dBRD9 treated cells.

Supplementary Table 2: List of primer sequences used for q-PCR.

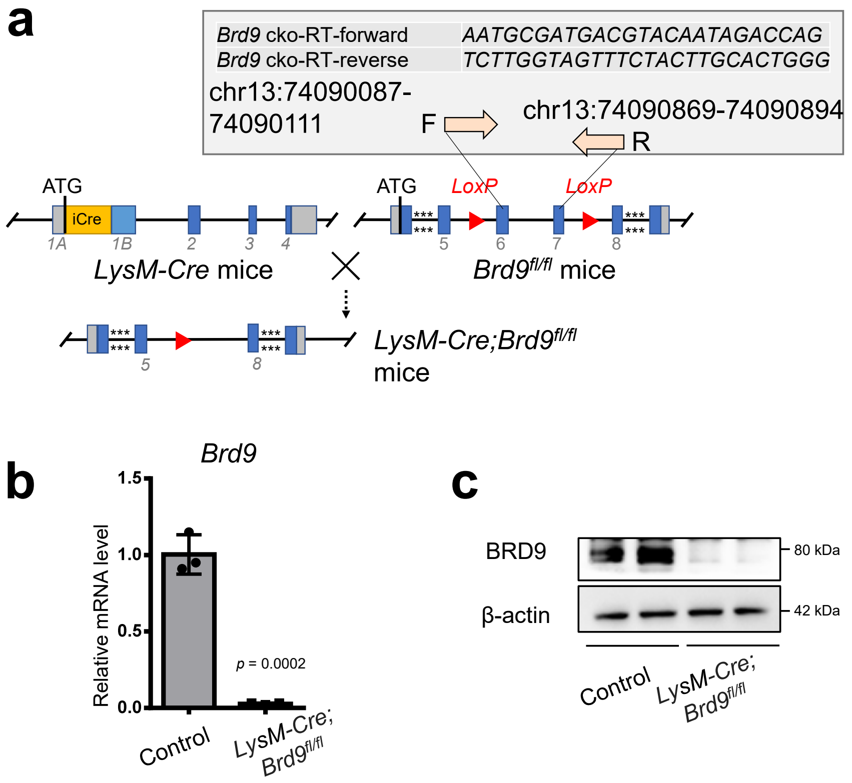

Supplementary Fig. 1 *Brd9* knockout strategy and efficiency in *LysM-Cre;Brd9$^{fl/fl}$* mice. a Illustration of *Brd9* deletion in *LysM*-expressing lineage. Mice bearing *loxP* sites encompassing the *Brd9 exon6-exon7* (*Brd9$^{fl/fl}$*) were crossed with those expressing Cre recombinase driven by the lysozyme M promoter (*LysM-Cre*). **b** *Brd9* mRNA expression in BMDMs derived from *LysM-Cre;Brd9$^{fl/fl}$* mice (n = 3) and littermate control mice (n = 3) after 3 days of RANKL induction, as measured by qPCR. **c** BRD9 protein expression in BMDMs derived from *LysM-Cre;Brd9$^{fl/fl}$* mice and littermate control mice after 3 days of RANKL induction, as measured by western blot. All data in this figure are represented as mean ± SD. Two-tailed Student's *t*-test for **b**. Source data are provided in the Source data file.

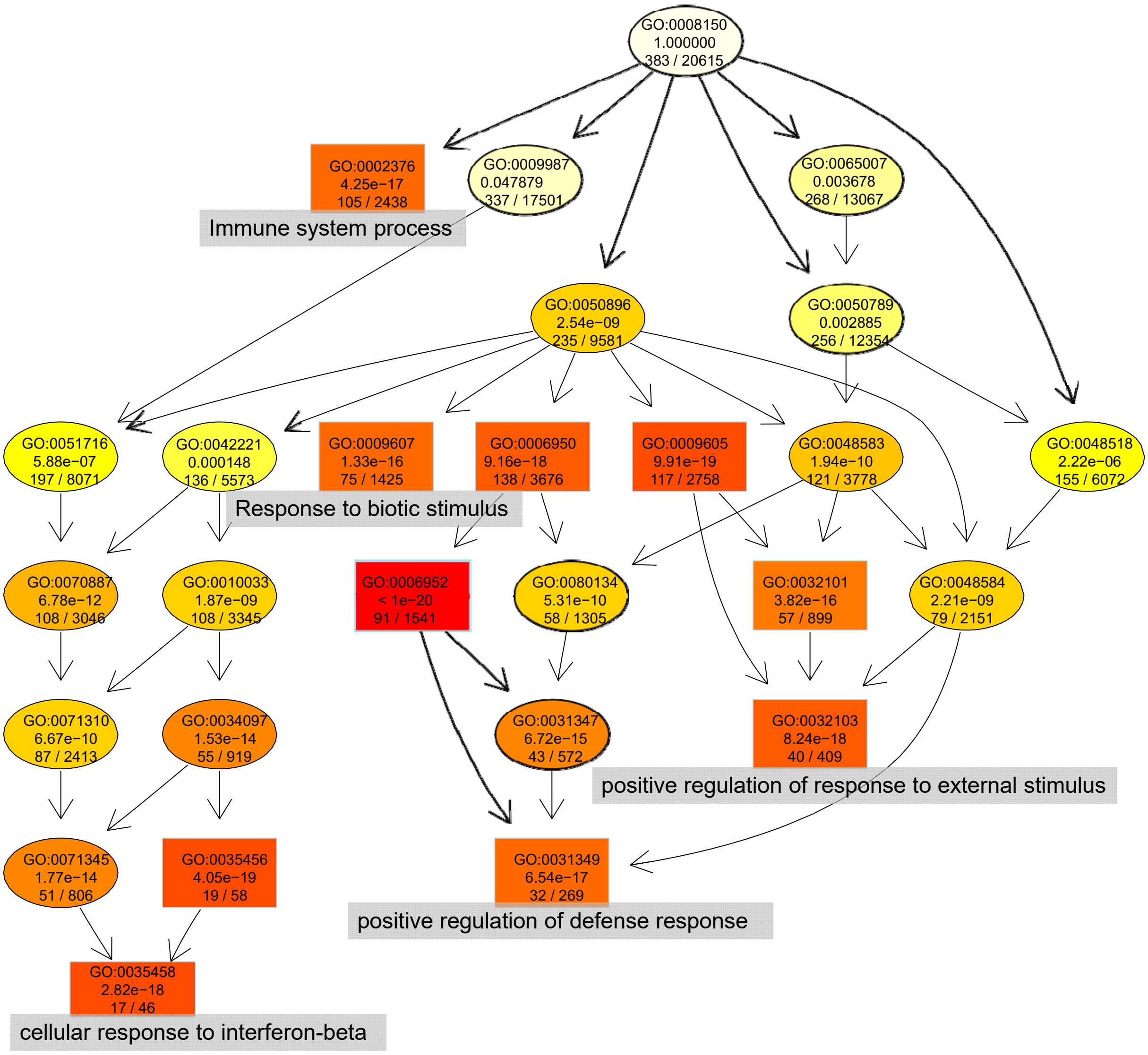

Supplementary Fig. 2 Directed acyclic graph of the top 10 GO terms for biological process in the comparison between BMDMs in MR+vector and MR+iBRD9 group. Every node indicates one GO term; boxes represent the top 10 enriched GO terms. *P* value under each GO term represents the enrichment degree, illustrated by color shades, with the darker the shade, the higher the enrichment.

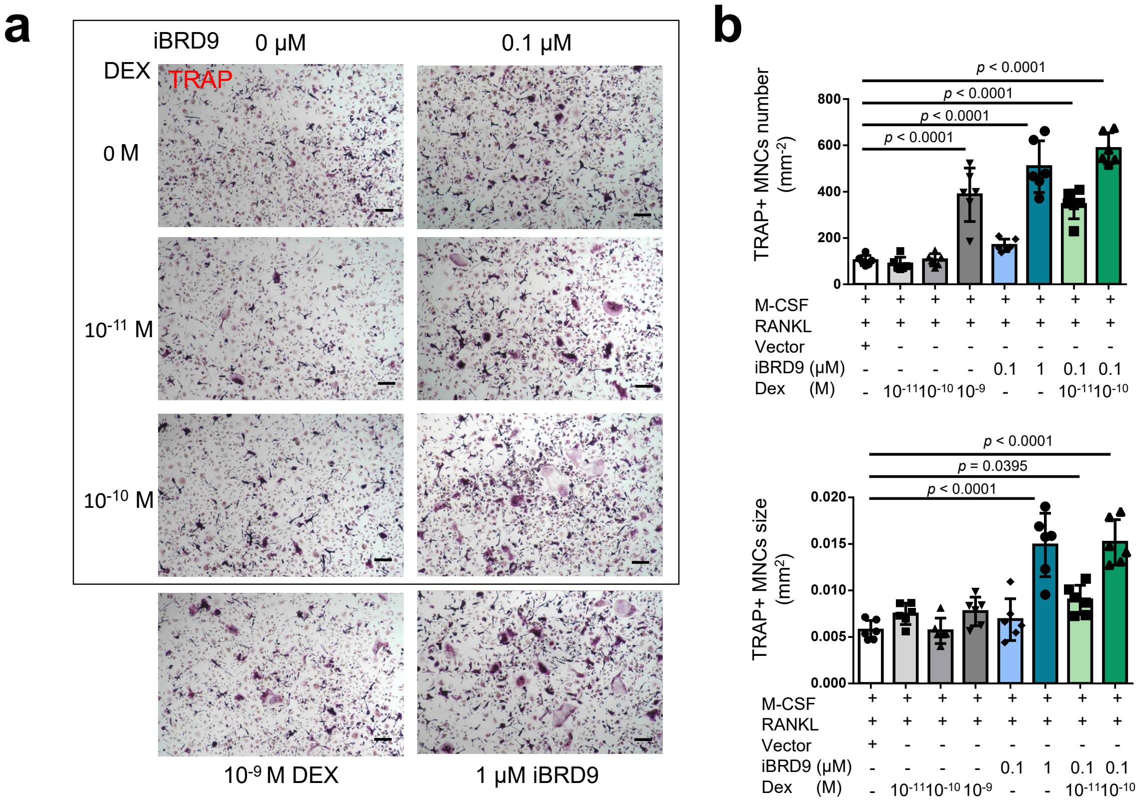

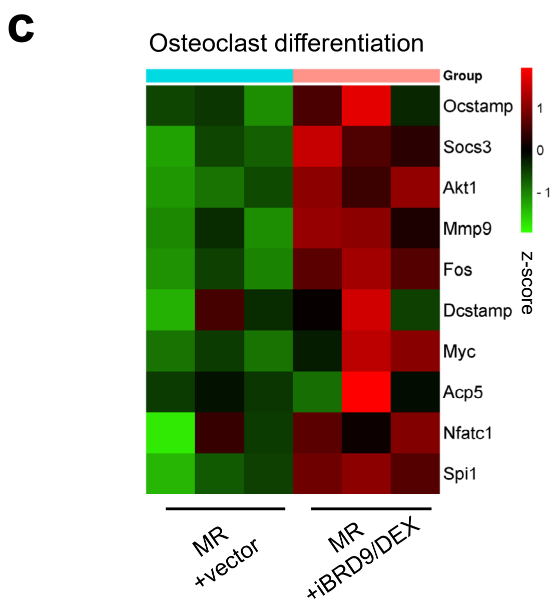

d

Term	Pathway	*P* value
GO:0008152	metabolic process	4.853E-12
GO:0044237	cellular metabolic process	2.576E-10
GO:0022900	electron transport chain	1.123E-09
GO:0043043	peptide biosynthetic process	2.139E-09
GO:0043604	amide biosynthetic process	3.3E-09
GO:0006518	peptide metabolic process	5.818E-09
GO:0006412	translation	6.258E-09
GO:0006091	generation of precursor metabolites and energy	2.22E-08
GO:0002181	cytoplasmic translation	3.278E-08
GO:1901566	organonitrogen compound biosynthetic process	3.957E-08

Supplementary Fig. 3 Synergizing function of BRD9 inhibition with glucocorticoid during osteoclastogenesis. a TRAP staining and **b** quantification analysis of BMDMs treated with 3 days of iBRD9 and/or DEX at different concentration during osteoclastic

induction. Scale bar, 200 μm. *n* = 6 biologically independent samples. **c** Heatmap showing the upregulated osteoclastic signature genes in 0.1 μM iBRD9/10^{-10} M DEX-treated group compared with the control group. Color scale represents normalized gene FPKM value by z-score scheme. **d** GO analysis shows the enriched top ten changed signaling pathway in 0.1 μM iBRD9/10^{-10} M DEX-treated group compared with the control group. All data in this figure are represented as mean ± SD. One-way analysis of variance (ANOVA) with Dunnett's multiple comparisons test for **b**. Hypergeometric distribution test for **d**. Source data are provided in the Source data file.

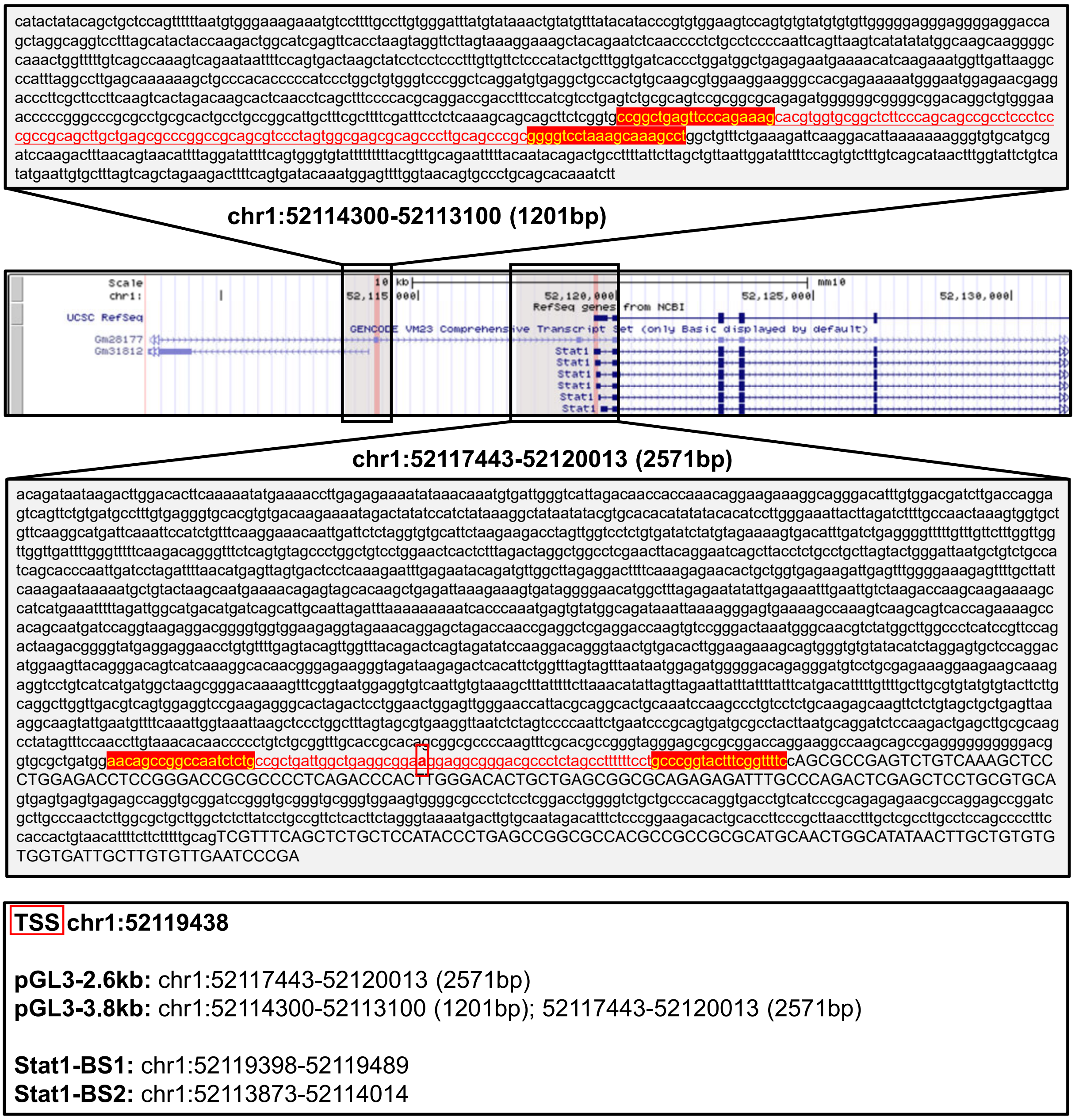

Supplementary Fig. 4 Genomic location of the region cloned in *Stat1* luciferase reporter plasmids and primer sets used in ChIP assay. Images representing the relative locations of the primer set amplicon (pink) at the promoter (2.6 kb, -1995 ~ +575, grey) and enhancer regions (1.2kb, -6338 ~ -5138, grey) of *Stat1* within the genome, as generated by the UCSC Genome Browser. The TSS is framed in red.

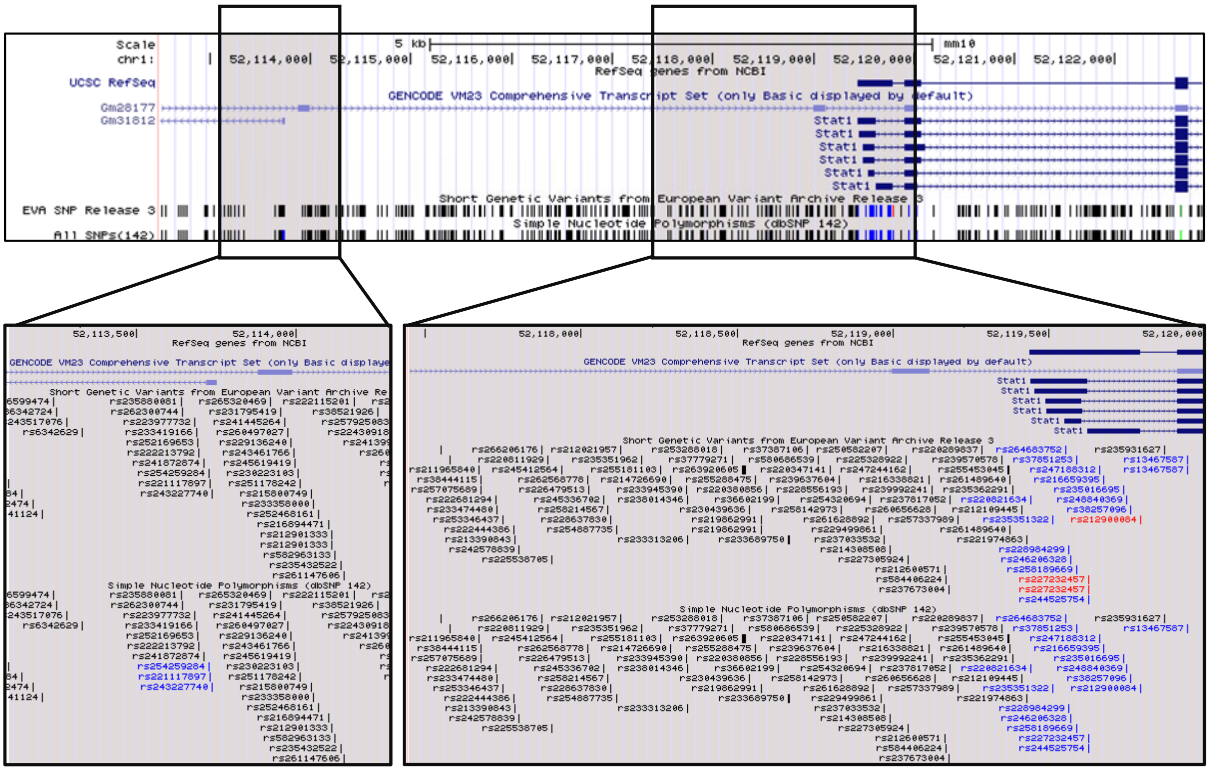

chr1:52114300-52113100 (1201bp) **chr1:52117443-52120013 (2571bp)**

Supplementary Fig. 5 Genomic location of the SNPs in predicted transcriptional regulatory region of *Stat1*. Images representing the relative locations of the SNPs at the promoter (2.6 kb, -1995 ~ +575) and enhancer (1.2kb, -6338 ~ -5138) regions of *Stat1* (grey region) within the genome, as generated by the UCSC Genome Browser. Red letters indicate protein-altering variants and splice site variants. Blue letters indicate non-coding transcript or untranslated region variants. Black letters indicate intergenic and intronic variants.

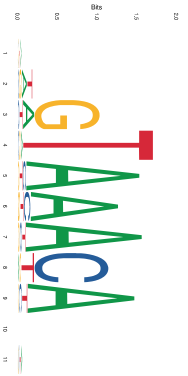

Matrix ID	Name	Score	Relative score	Sequence ID	Start	End	Strand	Predicted sequence
MA0481.3	MA0481.3.FOXP1	6.4241047	0.8195086100531784	seq1	68	78	+	gtataaactgt
MA0481.3	MA0481.3.FOXP1	7.8663597	0.8515891603141624	seq1	394	404	+	atgaaaacatc
MA0481.3	MA0481.3.FOXP1	11.95285	0.9424863083575683	seq1	1252	1262	+	atataaacaaa
MA0481.3	MA0481.3.FOXP1	5.7636924	0.8048188429468736	seq1	1374	1384	+	aagaaaataga
MA0481.3	MA0481.3.FOXP1	7.1026626	0.8346019948141608	seq1	1423	1433	+	atatatacaca
MA0481.3	MA0481.3.FOXP1	7.5115256	0.8436964697909778	seq1	1425	1435	+	atatacacatc
MA0481.3	MA0481.3.FOXP1	7.9726768	0.8539540050910539	seq1	1510	1520	+	aaggaaacaat
MA0481.3	MA0481.3.FOXP1	8.289424	0.8609995164333456	seq1	1941	1951	+	atgaaaacaga
MA0481.3	MA0481.3.FOXP1	8.36494	0.8626792379011531	seq1	2423	2433	+	gtgtatacatc
MA0481.3	MA0481.3.FOXP1	7.5264683	0.844028843955182	seq1	2659	2669	+	tcttaaacata
MA0481.3	MA0481.3.FOXP1	6.1368976	0.8131201679731789	seq1	2897	2907	+	tggtaaattaa

catactatacagctgctccagtttttaatgtgggaaagaaatgtccttttgccttgtgggatttat***gtataaactgt***atgtttatacatacccgtgtggaagtccagtgtgtatgtgtgttgggggagggaggggaggacca
gctaggcaggtcctttagcatactaccaagactggcatcgagttcacctaagtaggttcttagtaaaggaaagctacagaatctcaacccctctgcctccccaattcagttaagtcatatatatggcaagcaaggggc
caaactggtttttgtcagccaaagtcagaataattttccagtgactaagctatcctcctccctttgttgttctcccatactgctttggtgatcaccctggatggctgagagaatgaaaacatcaagaaatggttgattaaggc
ccatttaggccttgagcaaaaaagctgcccacaccccccatccctggctgtgggtcccggctcaggatgtgaggctgccactgtgcaagcgtggaaggaagggccacgagaaaaatgggaatggagaacgagg
acccttcgcttccttcaagtcactagacaagcactcaacctcagctttccccacgcaggaccgacctttccatcgtcctgagtctgcgcagtccgcggcgcagagatggggggcggggcggacaggctgtgggaa
acccccgggcccgcgcctgcgcactgcctgccggcattgctttcgcttttcgatttcctctcaaagcagcagcttctcggtgccggctgagttcccagaaagcacgtggtgcggctcttcccagcagccgcctccctcc
cgccgcagcttgctgagcgcccggccgcagcgtccctagtggcgagcgcagcccttgcagcccgcggggtcctaaagcaaagcctggctgtttctgaaagattcaaggacattaaaaaaagggtgtgcatgcg
atccaagactttaacagtaacattttaggatattttcagtgggtgtattttttttacgtttgcagaatttttacaatacagactgccttttattcttagctgttaattggatattttccagtgtctttgtcagcataactttggtattctgtca
tatgaattgtgctttagtcagctagaagacttttcagtgatacaaatggagttttggtaacagtgccctgcagcacaaatctt

chr1:52114300-52113100 (1201bp)

TSS chr1:52119438

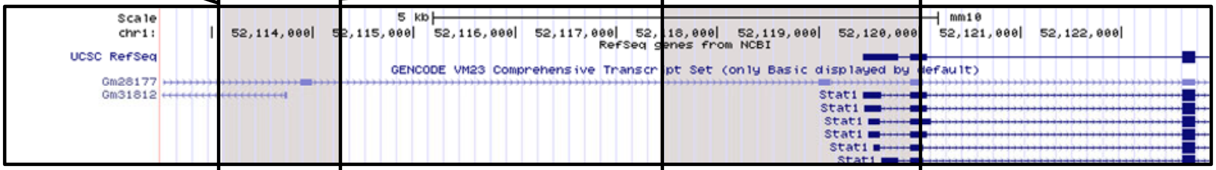

chr1:52117443-52120013 (2571bp)

acagataataagacttggacacttcaaaaatatgaaaaccttgagagaaa***atataaacaaa***tgtgattgggtcattagacaaccaccaaacaggaagaaaggcagggacatttgtggacgatcttgaccagga
gtcagttctgtgatgcctttgtgagggtgcacgtgtgac***aagaaaataga***ctatatccatctataaaggctataatatacgtgcacac***atatatacaca***tccttgggaaattacttagatcttttgccaactaaagtggtg
ctgttcaaggcatgattcaaattccatctgtttc***aaggaaacaat***tgattctctaggtgtgcattctaagaagacctagttggtcctctgtgatatctatgtagaaaagtgacatttgatctgaggggtttttgtttgttctttggtt
ggttggttgattttgggtttttcaagacagggtttctcagtgtagccctggctgtcctggaactcactctttagactaggctggcctcgaacttacaggaatcagcttacctctgcctgcttagtactgggattaatgctgtctgc
catcagcacccaattgatcctagattttaacatgagttagtgactcctcaaagaatttgagaatacagatgttggcttagaggacttttcaaagagaacactgctggtgagaagattgagtttggggaaagagttttgctt
attcaaagaataaaaatgctgtactaagca***atgaaaacaga***gtagcacaagctgagattaaagaaagtgataggggaacatggctttagagaatatattgagaaatttgaattgtctaagaccaagcaagaaaa
gccatcatgaaatttttagattggcatgacatgatcagcattgcaattagatttaaaaaaaaatcacccaaatgagtgtatggcagataaattaaaagggagtgaaaagccaaagtcaagcagtcaccagaaaag
ccacagcaatgatccaggtaagaggacggggtggtggaagaggtagaaacaggagctagaccaaccgaggctcgaggaccaagtgtccgggactaaatgggcaacgtctatggcttggccctcatccgttcc
agactaagacggggtatgaggaggaacctgtgttttgagtacagttggtttacagactcagtagatatccaaggacagggtaactgtgacacttggaagaaagcagtgggt***gtgtatacatc***taggagtgctccag
gacatggaagttacagggacagtcatcaaaggcacaacgggagaagggtagataagagactcacattctggtttagtagtttaataatggagatgggggacagagggatgtcctgcgagaaaggaagaagca
aagaggtcctgtcatcatgatggctaagcgggacaaaagtttcggtaatggaggtgtcaattgtgtaaagctttatttt***tcttaaacata***ttagttagaattatttattttatttcatgacattttgttttgcttgcgtgtatgtgtac
ttcttgcaggcttggttgacgtcagtggaggtccgaagagggcactagactcctggaactggagttgggaaccattacgcaggcactgcaaatccaagccctgtcctctgcaagagcaagttctctgtagctgctga
gttaaaggcaagtattgaatgttttcaaat***tggtaaattaa***gctccctggctttagtagcgtgaaggttaatctctagtccccaattctgaatcccgcagtgatgcgcctacttaatgcaggatctccaagactgagcttgc
gcaagcctatagtttccaaccttgtaaacacaacccccctgtctgcggtttgcaccgcacagcggcgccccaagtttcgcacgccgggtagggagcgcgcggaccaggaaggccaagcagccgagggggggg
ggacggtgcgctgatggaacagccggccaatctctgccgctgattggctgaggcggaaggaggcgggacgccctctagcctttttttcctgcccggtactttcggttttccAGCGCCGAGTCTGTCAAAG
CTCCCTGGAGACCTCCGGGACCGCGCCCCTCAGACCCACTTGGGACACTGCTGAGCGGCGCAGAGAGATTTGCCCAGACTCGAGCTCCTGCG
TGCAgtgagtgagtgagagccaggtgcggatccgggtgcgggtgcgggtggaagtggggcgccctctcctcggacctggggtctgctgcccacaggtgacctgtcatcccgcagagagaacgccaggagcc
ggatcgcttgcccaactcttggcgctgcttggctctcttatcctgccgttctcacttctagggtaaaatgacttgtgcaatagacatttctcccggaagacactgcaccttcccgcttaacctttgctcgccttgcctccagcc
cctttccaccactgtaacattttcttcttttttgcagTCGTTTCAGCTCTGCTCCATACCCTGAGCCGGCGCCACGCCGCCGCGCATGCAACTGGCATATAACTTGCTGT
GTGTGGTGATTGCTTGTGTTGAATCCCGA

Supplementary Fig. 6 FOXP1 motif hits in the predicted transcriptional regulatory region of *Stat1*. FOXP1 motif hits are indicated in italic bold letters in red, as generated by the UCSC Genome Browser and JASPAR. The TSS is framed in red.

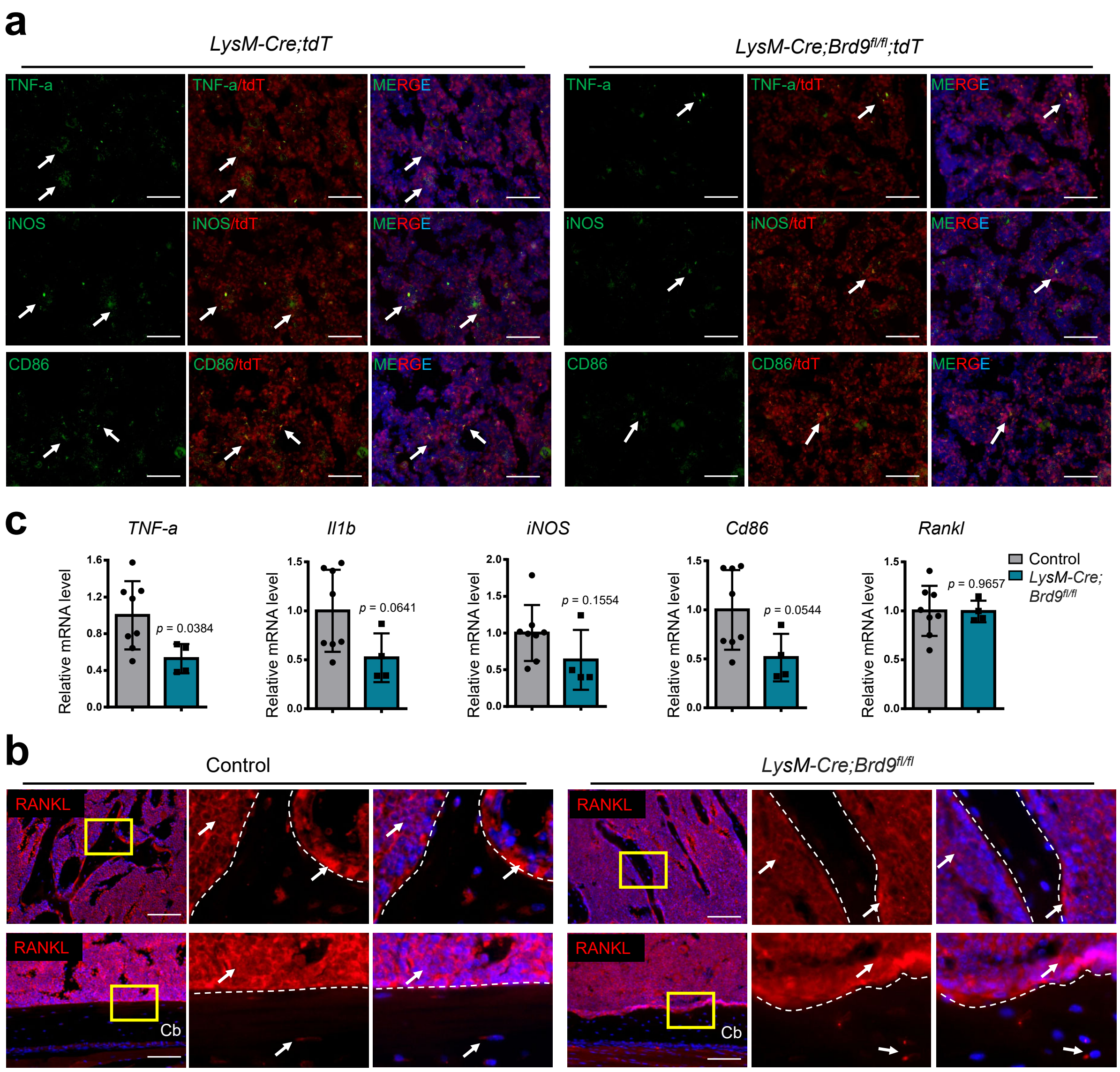

Supplementary Fig. 7 loss of *Brd9* led to slight inhibition on the basal expression of proinflammatory cytokines in the femur bone tissue. a TNF-α, iNOS and CD86 immunofluorescence (green) and visualization of tdTomato (red) of femurs from 4-week-old *LysM-Cre;Brd9$^{fl/fl}$;tdT* mice and littermate control mice. The progeny of the myeloid lineage shows red signal. Arrows indicate double positive signals. Scale bar, 100 μm. **b** immunofluorescence staining of RANKL (red) of femurs from 4-week-old *LysM-Cre;Brd9$^{fl/fl}$* mice and littermate control mice. Box in the left is shown at higher magnification in right. White dashed lines outline the bone surface. Arrows indicate positive signals. Cortical bone, Cb. Scale bar, 100 μm. **c** the mRNA expression of

proinflammatory cytokines of *TNF-α*, *Il1b*, *iNOS*, *Cd86*, and *Rankl* of femurs from 4-week-old *LysM-Cre;Brd9*$^{fl/fl}$ mice (*n* = 4) compared with that from control littermates (*n* = 8), as measured by qPCR. All data in this figure are represented as mean ± SD. Two-tailed Student's *t*-test for **c**. Source data are provided in the Source data file.

Supplementary Table 1: The top 10 GO biological process terms enriched in downregulated genes in dBRD9 treated cells. Hypergeometric distribution test used for GO analysis.

**Pathway ID**	**Pathway _ Down in MR+dBRD9**	***P* value**
GO:0035458	cellular response to interferon-beta	2.10306E-11
GO:0035456	response to interferon-beta	2.4753E-11
GO:0043170	macromolecule metabolic process	2.78763E-11
GO:0071310	cellular response to organic substance	4.30443E-11
GO:0048518	positive regulation of biological process	1.7978E-10
GO:0071704	organic substance metabolic process	8.52352E-10
GO:0044260	cellular macromolecule metabolic process	1.45113E-09
GO:0008152	metabolic process	2.10101E-09
GO:0009893	positive regulation of metabolic process	2.81374E-09
GO:0048522	positive regulation of cellular process	4.13079E-09

Supplementary Table 2: List of primer sequences used for q-PCR.

Gene	Forward	Reverse
β-actin	GGCTGTATTCCCCTCCATCG	CCAGTTGGTAACAATGCCATGT
Brd9 CKO	AATGCGATGACGTACAATAGACCAG	TCTTGGTAGTTTCTACTTGCACTGGG
Ctsk	CTCGGCGTTTAATTTGGGAGA	TCGAGAGGGAGGTATTCTGAGT
Foxp1	AAGTGTTTTGTGCGAGTAGAGAA	GGGAAGGGTTACCACTGATCTT
Mmp9	CTGGACAGCCAGACACTAAAG	CTCGCGGCAAGTCTTCAGAG
Acp5	CACTCCCACCCTGAGATTTGT	CATCGTCTGCACGGTTCTG
Brd9	TTGGAGATGGAAGTCTGCTCT	GCAACTTGCTAGACAGTGAACT
Dcstamp	GGGGACTTATGTGTTTCCACG	ACAAAGCAACAGACTCCCAAAT
Stat1	TCACAGTGGTTCGAGCTTCAG	GCAAACGAGACATCATAGGCA
Myc	ATGCCCCTCAACGTGAACTTC	CGCAACATAGGATGGAGAGCA
Fos	CGGGTTTCAACGCCGACTA	TTGGCACTAGAGACGGACAGA
iNOS	GTTCTCAGCCCAACAATACAAGA	GTGGACGGGTCGATGTCAC
TNF-a	CCCTCACACTCAGATCATCTTCT	GCTACGACGTGGGCTACAG
CD86	TGTTTCCGTGGAGACGCAAG	TTGAGCCTTTGTAAATGGGCA
Il6	TAGTCCTTCCTACCCCAATTTCC	TTGGTCCTTAGCCACTCCTTC
Il1b	GCAACTGTTCCTGAACTCAACT	ATCTTTTGGGGTCCGTCAACT
Rankl	CAGCATCGCTCTGTTCCTGTA	CTGCGTTTTCATGGAGTCTCA
