# Supplementary material for: BRD9-mediated chromatin remodeling suppresses osteoclastogenesis through negative feedback mechanism
Source: Nat Commun. 2023 Mar 14;14:1413. doi: 10.1038/s41467-023-37116-5 (PMC10014883; doi:10.1038/s41467-023-37116-5)
Supplement: Supplementary file 2 — Reporting Summary [file 41467_2023_37116_MOESM2_ESM.pdf]

nature portfolio

Corresponding author(s): Xinquan Jiang, Mingliang Zhou

Last updated by author(s): Feb 20, 2023

Reporting Summary

Nature Portfolio wishes to improve the reproducibility of the work that we publish. This form provides structure for consistency and transparency in reporting. For further information on Nature Portfolio policies, see our Editorial Policies and the Editorial Policy Checklist.

Statistics

For all statistical analyses, confirm that the following items are present in the figure legend, table legend, main text, or Methods section.

n/a	Confirmed	
☐	☒	The exact sample size (*n*) for each experimental group/condition, given as a discrete number and unit of measurement
☐	☒	A statement on whether measurements were taken from distinct samples or whether the same sample was measured repeatedly
☐	☒	The statistical test(s) used AND whether they are one- or two-sided<br>*Only common tests should be described solely by name; describe more complex techniques in the Methods section.*
☒	☐	A description of all covariates tested
☐	☒	A description of any assumptions or corrections, such as tests of normality and adjustment for multiple comparisons
☐	☒	A full description of the statistical parameters including central tendency (e.g. means) or other basic estimates (e.g. regression coefficient) AND variation (e.g. standard deviation) or associated estimates of uncertainty (e.g. confidence intervals)
☐	☒	For null hypothesis testing, the test statistic (e.g. *F*, *t*, *r*) with confidence intervals, effect sizes, degrees of freedom and *P* value noted<br>*Give P values as exact values whenever suitable.*
☒	☐	For Bayesian analysis, information on the choice of priors and Markov chain Monte Carlo settings
☒	☐	For hierarchical and complex designs, identification of the appropriate level for tests and full reporting of outcomes
☒	☐	Estimates of effect sizes (e.g. Cohen's *d*, Pearson's *r*), indicating how they were calculated

Our web collection on statistics for biologists contains articles on many of the points above.

Software and code

Policy information about availability of computer code

Data collection	NIS-Elements software (v4.5000.1117.0) and DP2-TWAIN software (v3.0.0.6212) were used to acquire images. Western blots were detected using UVITEC Alliance system (v16.0.3.0). Real-time reverse transcription PCR was performed using Roche LightCycler 480 system (v1.5.1.74). RNA-seq, ATAC-seq and ChIP-seq were conducted on Illumina NovaSeq 6000 platform.
Data analysis	CT data were reconstructed with NRecon software (v1.7.1.0, Bruker, Kontich, Belgium), and analyzed using the program CTAn (v1.16, Bruker, Kontich, Belgium).<br>Statistical analyses were performed with GraphPad Prism v6.01 software.<br>RNA-seq: Raw reads were filtered using Cutadapt (v1.15) and aligned with the GRCm39 genome using HISAT2 v2.0.5. Read Count values on each gene were compared using HTSeq (v0.9.1) and normalized to FPKM. Then difference expression of genes was analyzed using DESeq (v1.30.0) (fold change >= 1.2 and p < 0.05) and R language Pheatmap (v1.0.8) software package. GO enrichment analysis of the different expressed genes was performed using topGO (v2.40.0) (p < 0.05). The enrichment analysis of the KEGG pathway of differential genes was performed using ClusterProfiler (v3.4.4) software (p < 0.05). GSEA analysis was performed to functionally annotate the relevant genes and assess the enriched signaling pathways using GSEA_Linux_4.1.0.<br>ATAC-Seq: Library sequencing quality was assessed using Cutadapt (v1.9.1). Trimmed libraries were aligned to the GRCm39 mouse genome using Bowtie 2 (v2.2.6). PCR duplicates were removed and the number of mapped reads downsampled to a standard of ~20M using Picard (v1.126) (http://broadinstitute.github.io/picard/). ATAC-Seq peaks were called subsequently using MACS (v3.0.0a6) (-f BAMPE -B --SPMR –keep-dup all). Then the high-confidence set of peaks from all samples were merged to acquire consensus peakset. Potential DARs from consensus peakset were annotated with |log2(fold change)| >= 0.5. Motif analysis on DARs was performed by MEME (v5.4.1) suite with default settings with p < 0.0166. Gene ontology enrichment was performed using R package ClusterProfiler (v4.6.0), with default settings.<br>ChIP-seq: Raw reads were filtered using FASTX-Toolkit (v0.0.14) (http://hannonlab.cshl.edu/fastx_toolkit/). Clean reads were aligned to the GRCm39 mouse genome using Bowtie 2 (v2.3.5.1). Multiple aligned reads were filtered out using Picard (v2.27.5) (http://

broadinstitute.github.io/picard/). Then the unique mapped reads without duplicated reads were called for peaks using MACS2 (v2.2.71) with default parameters and $p < 0.05$. The peaks were annotated using ChIPseeker (v1.20.0) and motif analyses were carried out using the MEME (v4.1.2).

For manuscripts utilizing custom algorithms or software that are central to the research but not yet described in published literature, software must be made available to editors and reviewers. We strongly encourage code deposition in a community repository (e.g. GitHub). See the Nature Portfolio guidelines for submitting code & software for further information.

Data

Policy information about availability of data

All manuscripts must include a data availability statement. This statement should provide the following information, where applicable:

- Accession codes, unique identifiers, or web links for publicly available datasets
- A description of any restrictions on data availability
- For clinical datasets or third party data, please ensure that the statement adheres to our policy

The mRNA, ATAC and ChIP sequencing data generated in this study have been deposited in the Gene Expression Omnibus database under accession code GSE222240 [https://www.ncbi.nlm.nih.gov/geo/query/acc.cgi?acc=GSE222240]. GRCm39 genome is referenced in this study [http://asia.ensembl.org/Mus_musculus/Info/Index]. The other relevant data generated in this study are provided in the Supplementary Information/Source Data file.

Human research participants

Policy information about studies involving human research participants and Sex and Gender in Research.

Reporting on sex and gender	N/A
Population characteristics	N/A
Recruitment	N/A
Ethics oversight	N/A

Note that full information on the approval of the study protocol must also be provided in the manuscript.

Field-specific reporting

Please select the one below that is the best fit for your research. If you are not sure, read the appropriate sections before making your selection.

☒ Life sciences ☐ Behavioural & social sciences ☐ Ecological, evolutionary & environmental sciences

For a reference copy of the document with all sections, see nature.com/documents/nr-reporting-summary-flat.pdf

Life sciences study design

All studies must disclose on these points even when the disclosure is negative.

Sample size	No statistical methods were used to predetermine sample size. At least n = 3 mice of each groups were analyzed with exact sample size declared in corresponding figure legends. This was shown to be sufficient in previous studies, e.g. Deng, C. et al . Nat Commun 12, 2174 (2021); Sun, W. et al. Nat Commun 13, 2899 (2022); Jacome-Galarza, C.E. et al. Nature 568, 541–545 (2019), to discern statistically significant differences. In molecular biology experiments, n = 3 was chosen to generate p-values to determine if results are significant.
Data exclusions	No data were excluded in this study.
Replication	The in vitro experiments reported in the manuscript were replicated independently at least three times to confirm reproducibility. The exact sample size in terms of mice in all experiments are declared in corresponding figure legends.
Randomization	Samples and mice were randomly allocated to different groups.
Blinding	Analyses were done objectively. Blinding was not relevant for this study.

Reporting for specific materials, systems and methods

We require information from authors about some types of materials, experimental systems and methods used in many studies. Here, indicate whether each material, system or method listed is relevant to your study. If you are not sure if a list item applies to your research, read the appropriate section before selecting a response.

Materials & experimental systems

n/a	Involved in the study
☐	☒ Antibodies
☐	☒ Eukaryotic cell lines
☒	☐ Palaeontology and archaeology
☐	☒ Animals and other organisms
☒	☐ Clinical data
☒	☐ Dual use research of concern

Methods

n/a	Involved in the study
☐	☒ ChIP-seq
☒	☐ Flow cytometry
☒	☐ MRI-based neuroimaging

Antibodies

Antibodies used

Antibodies used in immunofluorescence staining as following: BRD9 antibody (Abcam, ab259839, 1:100), CTSK antibody (Abcam, ab37259, 1:100), STAT1 antibody (Cell signaling, 9172, 1:100), FOXP1 antibody (Merck Millipore, ABE68, 1:100), RUNX2 antibody (Thermo Fisher Scientific, MA5-41185, 1:100), iNOS (Abcam, ab178945, 1:100), TNF-a (Thermo Fisher Scientific, PA5-19810, 1:100), CD86 (Cell signaling, 19589, 1:100), RANKL antibody (bioworlde, BS72037, 1:100), Goat anti-Rabbit IgG (H+L) Cross-Adsorbed Secondary Antibody, Alexa Fluor 488 (Thermo Fisher Scientific, A-11008, 1:200), Donkey anti-Rabbit IgG (H+L) Highly Cross-Adsorbed Secondary Antibody, Alexa Fluor™ 594 (Thermo Fisher Scientific, A-21207, 1:200), Goat anti-Mouse IgG (H+L) Cross-Adsorbed Secondary Antibody, Alexa Fluor 488 (Thermo Fisher Scientific, A-11001, 1:200) and Goat anti-Mouse IgG (H+L) Cross-Adsorbed Secondary Antibody, Alexa Fluor 594 (Thermo Fisher Scientific, A-11005, 1:200).
Antibodies used in western blot as following: BRD9 antibody (Abcam, ab259839, 1:1000), MMP9 antibody (Abcam, ab228402, 1:1000), CTSK antibody (Abcam, ab37259, 1:1000), ACP5 antibody (Abcam, ab235448, 1:1000), β-actin antibody (Abcam, ab20272HRP, 1:5000), FOS antibody (Cell signaling, 4384, 1:1000), IFN-β1 antibody (Cell signaling, 97450, 1:1000), STAT2 antibody (Cell signaling, 72604, 1:1000), STAT1 antibody (Cell signaling, 9172, 1:1000), FOXP1 antibody (Cell signaling, 4402, 1:1000), Mouse IgG HRP-conjugated antibody (R&D, HAF007, 1:1000) and Rabbit IgG HRP-conjugated antibody (R&D, HAF008, 1:1000).
Anti-FOXP1 antibody (Cell signaling, 4402, 1:200) or normal Rabbit IgG (Cell Signaling, 2729, 1:4000) were used for immunoprecipitation. Immune complexes were subjected to immunoblotting with anti-FOXP1 (Cell signaling, 4402, 1:1000) or anti-BRD9 (Abcam, ab259839, 1:1000) antibodies.
Anti-FOXP1 antibody (Cell signaling, 4402, 1:100) with normal Rabbit IgG (Cell Signaling, 2729, 1:2000) as a non-specific IgG control, anti-BRD9 antibody (Bethyl Laboratories, A700-153, 1:100) with normal Rabbit IgG (Cell Signaling, 2729, 1:100) as a non-specific IgG control were used for chromatin immunoprecipitation.

Validation

All antibodies were commercial available and characterized by manufacturers online.
1. BRD9 antibody (https://www.abcam.cn/brd9-antibody-epr23888-5-ab259839.html);
2. CTSK antibody (https://www.abcam.com/cathepsin-k-antibody-3f9-ab37259.html);
3. STAT1 antibody (https://www.cellsignal.com/products/primary-antibodies/stat1-antibody/9172);
4. FOXP1 antibody (https://www.merckmillipore.com/CN/zh/product/Anti-FoxP1-Antibody,MM_NF-ABE68);
5. RUNX2 antibody (https://www.thermofisher.com/antibody/product/RUNX2-Antibody-clone-SD208-0-Recombinant-Monoclonal/MA5-41185);
6. iNOS antibody (https://www.abcam.com/iNOS-antibody-EPR16635-ab178945.html);
7. TNF-a antibody (https://www.thermofisher.com/antibody/product/TNF-alpha-Antibody-Polyclonal/PA5-19810?imageId=734330)1.
8. RANKL antibody (https://www.bioworlde.com/Primary-Antibodies/46043.html);
9. CD86 (https://www.cellsignal.cn/products/primary-antibodies/cd86-e5w6h-rabbit-mab/19589);
10. Goat anti-Rabbit IgG (H+L) Cross-Adsorbed Secondary Antibody, Alexa Fluor 488 (https://www.thermofisher.com/antibody/product/Goat-anti-Rabbit-IgG-H-L-Cross-Adsorbed-Secondary-Antibody-Polyclonal/A-11008);
11. Donkey anti-Rabbit IgG (H+L) Highly Cross-Adsorbed Secondary Antibody, Alexa Fluor™ 594 (https://www.thermofisher.com/antibody/product/Donkey-anti-Rabbit-IgG-H-L-Highly-Cross-Adsorbed-Secondary-Antibody-Polyclonal/A-21207);
12. Goat anti-Mouse IgG (H+L) Cross-Adsorbed Secondary Antibody, Alexa Fluor 488 (https://www.thermofisher.com/antibody/product/Goat-anti-Mouse-IgG-H-L-Cross-Adsorbed-Secondary-Antibody-Polyclonal/A-11001);
13. Goat anti-Mouse IgG (H+L) Cross-Adsorbed Secondary Antibody, Alexa Fluor 594 (https://www.thermofisher.com/antibody/product/Goat-anti-Mouse-IgG-H-L-Cross-Adsorbed-Secondary-Antibody-Polyclonal/A-11005);
14. β-actin antibody (https://www.abcam.com/hrp-beta-actin-antibody-mabcam-8226-loading-control-ab20272.html);
15. ACP5 antibody (https://www.abcam.cn/acid-phosphatase-antibody-epr21787-ab235448.html);
16. MMP9 antibody (https://www.abcam.com/mmp9-antibody-epr22140-154-ab228402.html);
17. FOS antibody (https://www.cellsignal.com/products/primary-antibodies/c-fos-antibody/4384);
18. IFN-β1 antibody (https://www.cellsignal.cn/products/primary-antibodies/ifn-b1-d2j1d-rabbit-mab-mouse-specific/97450);
19. STAT2 antibody (https://www.cellsignal.cn/products/primary-antibodies/stat2-d9j7l-rabbit-mab/72604);
20. Mouse IgG HRP-conjugated antibody (https://www.rndsystems.com/cn/products/mouse-igg-hrp-conjugated-antibody_haf007);
21. Rabbit IgG HRP-conjugated antibody (https://www.rndsystems.com/cn/products/rabbit-igg-hrp-conjugated-antibody_haf008);
22. Normal-rabbit-igg antibody (https://www.cellsignal.com/products/primary-antibodies/normal-rabbit-igg/2729);
23. BRD9 antibody (https://www.biomol.com/products/antibodies/primary-antibodies/general/anti-brd9-recombinant-monoclonal-a700-153-t?fs=1236343819).

Eukaryotic cell lines

Policy information about cell lines and Sex and Gender in Research

Cell line source(s)

RAW264.7 cell line was ordered from the cell bank of Cyagen Biosciences.

Authentication

The cell line has been validated using the short tandem repeat (STR) profiling method.

Mycoplasma contamination	The cell line has been tested negative for mycoplasma contamination.
Commonly misidentified lines (See ICLAC register)	No commonly misidentified cell lines were used.

Animals and other research organisms

Policy information about studies involving animals; ARRIVE guidelines recommended for reporting animal research, and Sex and Gender in Research

Laboratory animals	Brd9-flox mice (Strain NO. T008489), LysM-Cre mice (Strain NO. T003822) and WT C57BL/6J mice (Strain NO. N000013) and tdTomato (Strain NO.007909) mouse line were used and cross-bred in this study. LysM-Cre;Brd9fl/fl mice and control littermates at 4-week-old were analysed in this study. 6-week-old WT C57BL/6J mice were used for cell culture, zoledronate related osteonecrosis of the jaw and lipopolysaccharide-induced localized aggressive periodontitis. All mice were used for analysis regardless of sex. All mice were housed in pathogen-free conditions.
Wild animals	Our study did not involve wild animals.
Reporting on sex	All mice were used for analysis regardless of sex.
Field-collected samples	Our study did not involve samples collected from the field.
Ethics oversight	All animal studies were approved by the Institutional Animal Care and Use Committee at Ninth People's Hospital, School of Medicine, Shanghai Jiao Tong University (SH9H-2022-A926-1).

Note that full information on the approval of the study protocol must also be provided in the manuscript.

ChIP-seq

Data deposition

☒ Confirm that both raw and final processed data have been deposited in a public database such as GEO.

☒ Confirm that you have deposited or provided access to graph files (e.g. BED files) for the called peaks.

Data access links *May remain private before publication.*	https://www.ncbi.nlm.nih.gov/geo/query/acc.cgi?acc=GSE222240
Files in database submission	GSM6934376 MR<br>GSM6934377 M_Input
Genome browser session (e.g. UCSC)	No longer applicable.

Methodology

Replicates	ChIP-seq experiments were performed in singlicate per condition per experiment.
Sequencing depth	Double-end sequencing was used with 150 bp read length.<br>MR, uniquely mapped reads: 48894980, called peaks: 20236<br>M_Input, input reads: 28203811, called peaks: na
Antibodies	Anti-BRD9 antibody (Bethyl Laboratories, A700-153)
Peak calling parameters	Clean reads were aligned to the GRCm39 mouse genome using Bowtie 2 (v2.3.5.1). Multiple aligned reads were filtered out using Picard (v2.27.5) (http://broadinstitute.github.io/picard/). Then the unique mapped reads without duplicated reads were called for peaks using MACS2 (v2.2.71) with default parameters and p < 0.05. The peaks were annotated using ChIPseeker (v1.20.0) and motif analyses were carried out using the MEME (v4.1.2).
Data quality	Raw reads were filtered using FASTX-Toolkit (v0.0.14) (http://hannonlab.cshl.edu/fastx_toolkit/).MR, called peaks: 20236, p-value < 0.05
Software	FASTX-Toolkit (v0.0.14), Bowtie 2 (v2.3.5.1), Picard (v2.27.5), MACS2 (v2.2.71), ChIPseeker (v1.20.0), MEME (v4.1.2)
